# Supplementary material for: LOTUS overexpression via ex vivo gene transduction further promotes recovery of motor function following human iPSC-NS/PC transplantation for contusive spinal cord injury
Source: Stem Cell Reports. 2021 Oct 14;16(11):2703–17. doi: 10.1016/j.stemcr.2021.09.006 (PMC8580872; doi:10.1016/j.stemcr.2021.09.006)
Supplement: Document S1. Supplemental experimental procedures and Figures S1 and S2 [file mmc1.pdf]

Stem Cell Reports, Volume *16*

Supplemental Information

LOTUS overexpression via *ex vivo* gene transduction further promotes recovery of motor function following human iPSC-NS/PC transplantation for contusive spinal cord injury

Shuhei Ito, Narihito Nagoshi, Yasuhiro Kamata, Kota Kojima, Satoshi Nori, Morio Matsumoto, Kohtaro Takei, Masaya Nakamura, and Hideyuki Okano

Supplementary Information

LOTUS overexpression via lentiviral *ex vivo* gene transduction further promotes recovery of motor function following human iPSC-NS/PC transplantation for contusive spinal cord injury

Shuhei Ito, Narihito Nagoshi, Yasuhiro Kamata, Kota Kojima, Satoshi Nori, Morio Matsumoto, Kohtaro Takei, Masaya Nakamura, and Hideyuki Okano

Figure S1

A.

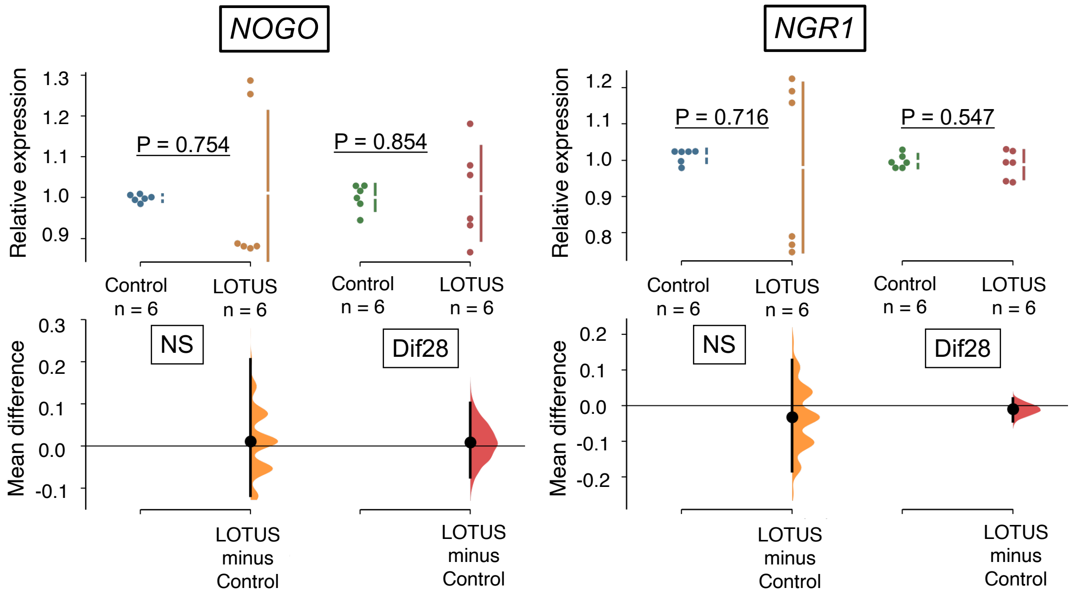

Figure S1

Gene expression change of LOTUS-expressing NS/PCs in *NOGO* and *NGR1* in neurospheres and differentiated cells derived from 414C2 hiPSC-NS/PCs.

***A*,** Quantitative real time PCR analyses for the gene expression of *NOGO* and *NGR1* in both NS/PC groups (LOTUS-NS/PCs; n = 6 independent experiments, Control-NS/PCs; n = 6 independent experiments). Values are the mean ± SEM; *p < 0.05, **p < 0.01. Statistical analysis was performed using the Mann–Whitney U test in each real time PCR analysis.

Figure S2

A.

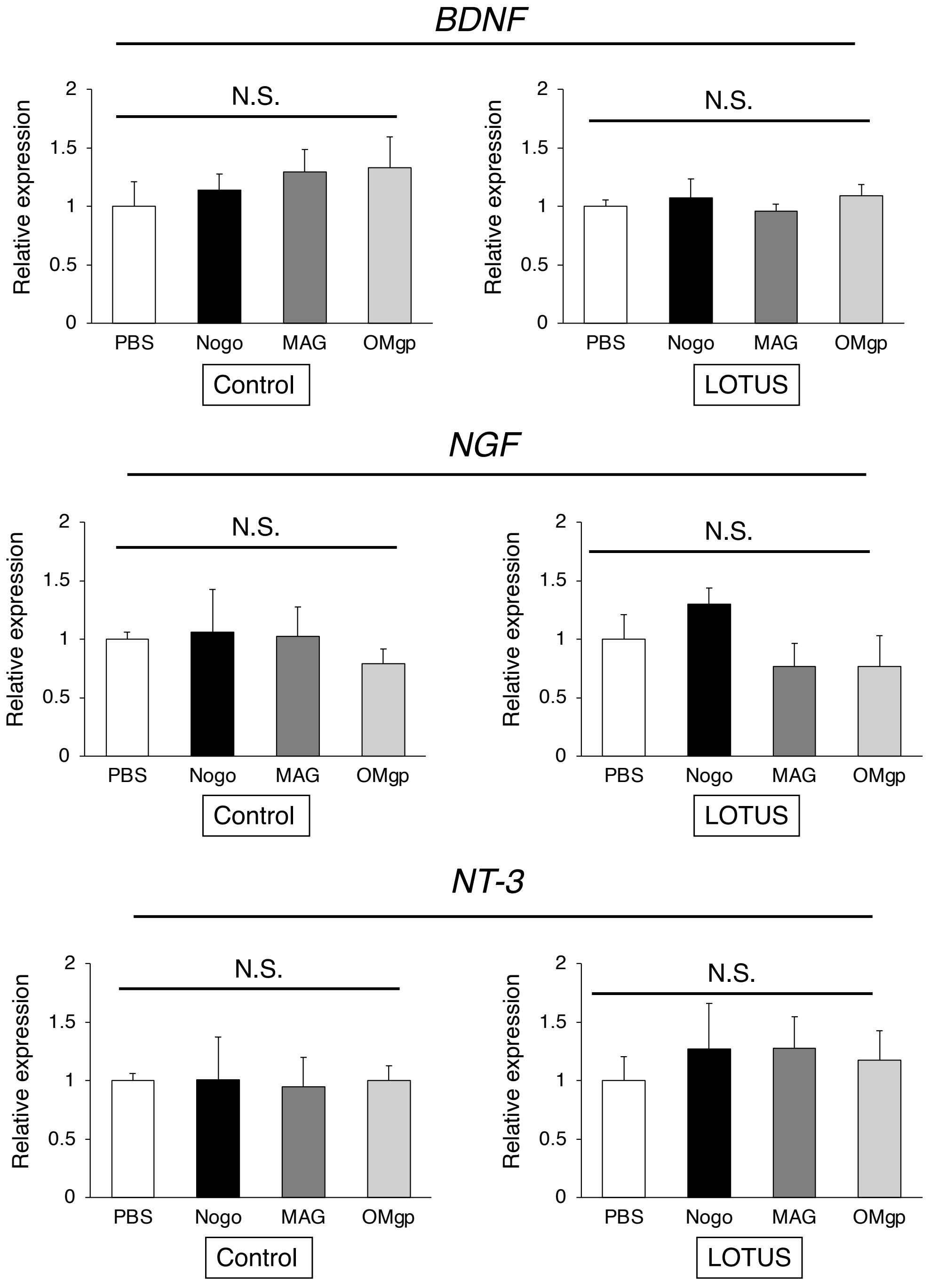

Figure S2

Gene expression change of the Control-NS/PCs and LOTUS-NS/PCs in the neurotrophic factors under the presence of NgR1 ligands.

A, Quantitative real time PCR analyses for the gene expression of *BDNF, NGF* and *NT-3* under precoated with PBS, Nogo, MAG or OMgp in both NS/PC groups (LOTUS-

NS/PCs; n = 4 independent experiments, Control-NS/PCs; n = 4 independent experiments). Values are the mean ± SEM; *$p < 0.05$, **$p < 0.01$. Statistical analysis was performed using one-way ANOVA followed by the Tukey-Kramer test in each real time PCR analysis.

Supplemental Experimental Procedures

Cell culture, lentivirus transduction, and neuronal differentiation analysis

The cell culture and neural induction of hiPSCs (414C2) were performed as previously described (Itakura et al., 2015) with slight modifications. The hiPSCs were cultured for 12 days on gelatin-coated (0.1%) culture dishes with mouse embryonic fibroblasts and then formed into embryo bodies in floating culture for 30 days. Aggregated cells were dissociated into single cells using TrypLE Select (Thermo Fisher Scientific, Yokohama, Japan) and cultured in media containing a hormone mixture supplemented with B27 and 20 ng/ml FGF-2 (Pepro Tech, Rocky Hill, NJ) and 10 ng/ml human leukemia inhibitory factor (hLIF; Merck KGaA, Darmstadt, Germany) for 12 days. These primary neurospheres were passaged to fourth neurospheres for the *in vitro* experiment and transplantation.

LOTUS-NS/PCs were prepared via lentivirus transduction. The 414C2-hiPSC-derived primary neurospheres were dissociated and infected with the mLOTUS-expressing lentivirus. Subsequently, these secondary neurospheres were dissociated and infected with lentivirus to express ffLuc, a fusion protein between Venus fluorescent protein and firefly luciferase (Hara-Miyauchi et al., 2012) under the control of the EF1α promoter (pCSII-EF1α-ffLuc). These secondary neurospheres were passaged into tertiary and fourth neurospheres and used for *in vitro* analysis and transplantation. In contrast, the Control-NS/PCs were prepared by similar transduction via lentivirus expressing ffLuc and used for the analyses.

Neuronal differentiation analysis was performed as follows. Dissociated hiPSC-NS/PCs were plated onto poly-D-lysin/laminin-coated 48-well slides (Costar 3548; Corning, NY, USA) at a density of 1×10^5 cells/ml and cultured in medium without growth factors at 37 °C in 5% CO_2 and 95% air for 2, 14 and 28 days. Differentiated cells were fixed with 4% paraformaldehyde (PFA) in 0.1 M phosphate buffered saline (PBS) for 15 minutes at RT and stained with the following primary antibodies for immunocytochemistry: anti-GFP (goat IgG, 1:500, Rockland, Ireland), anti-MAP2 (rabbit IgG, 1:500, Merck Millipore, Billerica, Massachusetts, USA) and anti-cleaved caspase-3 (rabbit IgG, 1:500, Cell Signaling, Beverly, MA, USA). Then, the sections were incubated with Alexa Fluor-conjugated secondary antibodies (1:1000). Nuclei were stained with Hoechst 33258 (10

µg/ml, Sigma-Aldrich, St. Louis, MO, USA). All *in vitro* images were obtained using confocal laser scanning microscopy (LSM 700; Carl Zeiss, Jena, Germany).

SCI and transplantation

Previous study (Nori et al., 2011) showed the sufficient functional recovery of BMS 4-5 following cell transplantation alone. In this study, we compared three groups: PBS, Control-NS/PCs and LOTUS-NS/PCs. Even though the efficacy of cell transplantation alone has been demonstrated, these experiments were conducted to show the efficacy of cell transplantation with ex-vivo gene transduction. To prevent ceiling effect of the locomotor function, we induced severer model of SCI with 65kdyn.

Adult female NOD-SCID mice were anesthetized by intraperitoneal injections of ketamine (60 mg/kg) and xylazine (10 mg/kg). The laminal arch of the vertebrae at the tenth thoracic level was removed, and the dorsal surface of the dura mater was exposed and subjected to a 65-kdyn contusive SCI using a commercially available SCI device (IH impactor, Precision Systems and Instrumentation) as previously described(Scheff et al., 2003). After SCI, 12.5 mg/kg ampicillin was administered intramuscularly. Nine days after the injury, 5×10^5 LOTUS-NS/PCs/2 µl were transplanted into the lesion epicenter of each mouse (LOTUS group; n = 18) using a metal needle at a rate of 1 µl/minute with a 10 µl Hamilton syringe and a stereotaxic microinjector (KDS 310; Muromachi Kikai, Tokyo, Japan). Using the same method, 5×10^5 Control-NS/PC/2 µl were injected into each mouse (control group; n = 17). An equal volume of PBS was injected into the PBS group of mice (n = 12).

Western blotting

We performed western blotting to determine the protein expression of the Control-NS/PCs and the LOTUS-NS/PCs. The fourth passage neurospheres that underwent differentiation for 14 days were used for analysis. Cell samples were homogenized in lysis buffer, and 10 ng protein was electrophoretically separated on 4–20% gradient polyacrylamide gels and transferred onto polyvinylidene difluoride (PVDF) membranes. The membranes were blocked with 5% skim milk in Tris-buffered saline and 0.1% Tween 20 (TBST) for 1 h at room temperature (RT) and then incubated overnight at 4 °C with an affinity-purified monoclonal mouse antibody against LOTUS (ITM). After the membranes were washed with TBST, they were incubated with horseradish peroxidase-

conjugated secondary antibody for 1 h at RT. The bands were visualized using an enhanced chemiluminescence reagent (GE Healthcare) and an ImageQuant LAS 4000 instrument (GE Healthcare).

Real-time PCR

Total RNA was extracted from hiPSC-NS/PCs (LOTUS-NS/PCs; n = 5, Control-NS/PCs; n = 5) by using an RNeasy Micro Kit (Qiagen, Inc., Hilgen, Germany), and cDNA was synthesized by reverse transcription with ReverTra Ace qPCR RT master mix (ToyoboCo., Ltd., Life Science Department, Osaka, Japan). Quantitative polymerase chain reaction (QT-PCR) was performed using Step One Plus™ (Applied Biosystems, Foster City, CA, USA) following the manufacturer's instructions. The expression levels of each gene were normalized to that of GAPDH using the ΔΔCT method. We chose primers used for the TaqMan gene expression assays and TaqMan gene expression 96-well fast plates (Life Technologies, Carlsbad, CA, USA). We used the following manufactured primers (Applied Biosystems) against mouse or human DNA sequences, such as *Lotus, MAP2, GFAP, OLIG2, NNESTIN, BDNF, NGF, NT-3 and GAPDH.*

Neurite outgrowth assay on NgR1 ligand-coated plates

NgR1 ligand-coated plates were prepared for the neurite outgrowth assay. Twenty-four-well chamber slides (Costar 3524; Corning, NY, USA) were coated with poly-D-lysin/laminin overnight at 37 °C in 5% CO2 and 95% air. These slides were washed with PBS and dried for 30 minutes. Then, Nogo-66-Fc (400 nM, R&D Systems), MAG-AP (300 nM, R&D Systems) or OMgp (200 nM, R&D Systems) was added to the slide and incubated at 37 °C for 2 h. After washing, these plates were coated with laminin overnight at 37 °C in 5% CO2 and 95% air. Dissociated hiPSC-NS/PCs were plated onto NgR1 ligand-coated plates at a density of 1.2×10^4 cells/ml and cultured in medium without growth factors at 37 °C in 5% CO2 and 95% air for 2 and 14 days. Immunostaining was performed with an anti-MAP2 antibody in blocking solution overnight at 4 °C, followed by incubation with Alexa555-labeled goat antibodies against rabbit IgG (1:1000 dilution, Invitrogen) in blocking solution for 1 h at RT. Nuclei were stained with Hoechst 33258. The images were obtained using a confocal laser scanning microscope (LSM 700; Carl Zeiss, Jena, Germany) with 20x objective lenses. We randomly obtained five regions in the culture slides, and then neurite length of the differentiated NS/PCs was measured by

manually tracing the length of the longest neurite per cell (using NIH Image software). Five longest neurites of each cell on each region were measured (n = 5 in the both hiPSC-NS/PCs).

Apoptosis analysis

Twenty-four-well chamber slides (Costar 3524; Corning, NY, USA) were coated with poly-D-lysin/laminin overnight at 37 °C in 5% CO2 and 95% air, followed by washing with PBS and drying for 30 minutes. Nogo-66-Fc (400 nM, R&D Systems) was added to the slide and incubated at 37 °C for 2 h. After washing, these plates were coated with laminin overnight at 37 °C in 5% CO2 and 95% air. Dissociated hiPSC-NS/PCs were plated onto the Nogo-coated plates at a density of 1.2×10^4 cells/ml and cultured in medium without growth factors at 37 °C in 5% CO2 and 95% air for 2 days. Immunostaining was performed with anti-cleaved caspase-3 in blocking solution overnight at 4°C, followed by incubation with Alexa555-labeled goat antibodies against rabbit IgG (1:1000 dilution, Invitrogen) in blocking solution for 1 h at RT. Nuclei were stained with Hoechst 33258. The images were obtained using confocal laser scanning microscopy (LSM 700) with 20x objective lenses. We randomly obtained five images in the culture slides,and then the cleaved caspase-3-positive apoptotic cells and nuclei were counted in each image.These counts were expressed as a percentage of the total nuclei (*n* =5, each group).

***In vivo* imaging of transplanted cells**

For confirmation of the survival of the transplanted hiPSC-NS/PCs, a Xenogen-IVIS spectrum cooled charge-coupled device (CCD) optical macroscopic imaging system (Caliper Life-Science, Hopkinton, MA, USA) was used for bioluminescence imaging (BLI). Monitoring was performed once per week following cell transplantation. Six mice in Control group and eight mice in LOTUS group were examined for *in vivo* imaging. In brief, D-luciferin (VivoGlo Luciferin; Promega, Madison, WI) was intraperitoneally injected at a dose of 300 mg/kg body weight. Animals were placed in a light-tight chamber, and photons emitted from luciferase-expressing cells were collected with integration times of 5 s to 2 minutes, depending on the intensity of bioluminescence emission. BLI signals were quantified in maximum radiance units (photons per second per centimeter squared per steradian (p/s/cm2/sr)) and are presented as log10 (photons per second) values.

Immunohistochemistry (IHC)

Anesthetized mice were transcardially perfused with heparinized saline solution, followed by 4% paraformaldehyde PBS 63 days after injury. Spinal cords were dissected and postfixed in 4% paraformaldehyde for 2 h at RT. Fixed spinal cords were soaked in 10% sucrose in 0.1 M PBS overnight at 4 °C, followed by 30% sucrose, embedding in Optimal Cutting Temperature compound (Sakura FineTechnical Co., Ltd.), and freezing as previously described(Nishimura et al., 2013). Samples were sectioned in the sagittal plane at a thickness of 14 µm or the axial plane at a thickness of 20 µm on a cryostat (Leica CM3050 S, Leica Microsystems). Histological analyses of the sections were performed by hematoxylin-eosin (HE) staining and IHC. Tissue sections were stained with the following primary antibodies for IHC: anti-STEM121 (mouse IgG1, 1:100; TaKaRa Bio, Y40410), anti-Oct4 (mouse IgG1, 1:50 Santa Cruz, sc-5279), anti-Ki67 (rabbit IgG, 1:200; Abcam, ab15580), anti- human Nestin (rabbit IgG, 1:200; Immuno-Biological Laboratories, Takasaki, Japan, 18741), anti-HuC/HuD (ELAVL3/4) (mouse IgG2, 1:100 Invitrogen, A21271), anti-glial fibrillary acidic protein (GFAP; rabbit IgG, 1:500; Dako, Z0334), anti-APC (mouse IgG2b, 1:300; Abcam, ab16794), anti-neurofilament 200 kDa (NF-H; mouse IgG, 1:500; Millipore, MAB5262), anti-5-HT (goat IgG, 1:500; Immunostar, Inc., 20079), anti-b-tubulin-3 (mouse IgG2b, 1:500), anti-nuclei (HNA, mouse IgG1; Sigma-Aldrich, 1:200, MAB4383), anti-Bsn (mouse IgG, 1:200; Stressgen, ADI-VAM-PS003), and anti-hSyn (mouse IgG, 1:200; Merck KGaA, MAB332). Then, the sections were incubated with Alexa Fluor-conjugated secondary antibodies (1:1000). Nuclei were stained with Hoechst 33258. All images were obtained using a confocal laser scanning microscope (LSM 700, Carl Zeiss).

Quantitative analysis of the tissue sections

The spinal cord area was quantified using HE staining of axial sections at the lesion epicenter and 4 mm rostral and caudal to the epicenter under a 10x objective (n = 5, each group). Quantitative analysis of the STEM121 positive fibers was performed using the immunoreactive areas in midsagittal sections at the lesion epicenter and 4 mm rostral and caudal to the epicenter under a 40x objective (n = 5, each group). To quantify the NF-H-positive fibers, the midsagittal sections were captured at 4 mm caudal to the epicenter under a 40x objective (n = 5, each group). To quantify the 5-HT-positive fibers,

we scanned the ventral horn of axial sections at 4 mm caudal to the epicenter (lumbar enlargement) under a 20x objective (*n* = 5, each group). To quantify the proportion of each cell phenotype in vivo, we selected representative midsagittal sections and randomly obtained five regions in the graft cells under a 20x objective (*n* = 5, each group). The numbers of marker-positive cells, such as HNA-, Oct4-, Ki67-, Nestin-, ELAVL3/4 (HuC/D)-, GFAP-, and APC-positive cells were counted in each section (n = 5 per group). All images were quantified using ImageJ (https://imagej.nih.gov/ij/). The threshold values were maintained at a constant level for all analyses using ImageJ.

Behavioral analysis

The hindlimb locomotor function of each mouse was evaluated weekly using the Basso mouse scale (BMS) up to 63 days after injury (54 days after transplantation)(Basso et al., 2006). The BMS scores was determined for 13 mice in Control group, 15 mice in LOTUS group and 10 mice in PBS group. Two persons blinded to the mouse group performed the behavioral analyses. At 63 days after injury, motor function was also assessed on a rotating rod(Rotarod)apparatus (KDS310; Muromachi-Kikai Co., Ltd.) by measuring the amount of time that mice could remain on the rod while it rotated at 10 rotations per minute (rpm). Three trials were conducted and the average number of seconds was recorded. Six mice each from the three groups were examined in the Rotarod test. A treadmill gait analysis was performed using the DigiGait System (Mouse Specifics). Ten mice each from control group and LOTUS group and 5 mice from PBS group were used. Three cycles of continuous gait were recorded and each parameters of each limb were measured. The stride lengths and stance angles of the hindlimbs were measured on a treadmill at a speed of 7 cm/s, and phase dispersion was analyzed in the DigiGait analyses to provide an indication of limb coordination.

Supplemental Reference

Nishimura S, Yasuda A, Iwai H, Takano M, Kobayashi Y, Nori S, Tsuji O, Fujiyoshi K, Ebise H, Toyama Y, Okano H, Nakamura M (2013) Time-dependent changes in the microenvironment of injured spinal cord affects the therapeutic potential of neural stem cell transplantation for spinal cord injury. Mol Brain 6:3.
