# Supplementary material for: LOTUS overexpression via ex vivo gene transduction further promotes recovery of motor function following human iPSC-NS/PC transplantation for contusive spinal cord injury
Source: Stem Cell Reports. 2021 Oct 14;16(11):2703–17. doi: 10.1016/j.stemcr.2021.09.006 (PMC8580872; doi:10.1016/j.stemcr.2021.09.006)
Supplement: Document S2. Article plus supplemental information [file mmc2.pdf]

Stem Cell Reports
Article
OPEN ACCESS

LOTUS overexpression via *ex vivo* gene transduction further promotes recovery of motor function following human iPSC-NS/PC transplantation for contusive spinal cord injury

Shuhei Ito,[1,2,3] Narihito Nagoshi,[1] Yasuhiro Kamata,[1] Kota Kojima,[1] Satoshi Nori,[1] Morio Matsumoto,[1] Kohtaro Takei,[4] Masaya Nakamura,[1,*] and Hideyuki Okano[2,*]
[1]Department of Orthopaedic Surgery, Keio University School of Medicine, 35 Shinanomachi, Shinjuku-ku, Tokyo 160-8582, Japan
[2]Department of Physiology, Keio University School of Medicine, 35 Shinanomachi, Shinjuku-ku, Tokyo 160-8582, Japan
[3]Department of Orthopaedic Surgery, National Hospital Organization Tokyo Medical Center, 2-5-1 Higashigaoka, Meguro-ku, Tokyo 152-8902, Japan
[4]Molecular Medical Bioscience Laboratory, Yokohama City University Graduate School of Medical Life Science, 1-7-29 Suehirocho, Tsurumi-ku, Yokohama, Kanagawa 230-0045, Japan
*Correspondence: masa@keio.jp (M.N.), hidokano@a2.keio.jp (H.O.)
https://doi.org/10.1016/j.stemcr.2021.09.006

SUMMARY

Functional recovery is still limited mainly due to several mechanisms, such as the activation of Nogo receptor-1 (NgR1) signaling, when human induced pluripotent stem cell-derived neural stem/progenitor cells (hiPSC-NS/PC) are transplanted for subacute spinal cord injury (SCI). We previously reported the neuroprotective and regenerative benefits of overexpression of lateral olfactory tract usher substance (LOTUS), an endogenous NgR1 antagonist, in the injured spinal cord using transgenic mice. Here, we evaluate the effects of lentiviral transduction of LOTUS gene into hiPSC-NS/PCs before transplantation in a mouse model of subacute SCI. The transduced LOTUS contributes to neurite extension, suppression of apoptosis, and secretion of neurotrophic factors *in vitro*. *In vivo*, the hiPSC-NS/PCs enhance the survival of grafted cells and enhance axonal extension of the transplanted cells, resulting in significant restoration of motor function following SCI. Therefore, the gene transduction of LOTUS in hiPSC-NS/PCs could be a promising adjunct for transplantation therapy for SCI.

INTRODUCTION

Several studies have reported the efficacy of human induced pluripotent stem cell-derived neural stem/progenitor cell (hiPSC-NS/PC) transplantation at the subacute stage of spinal cord injury (SCI) (Fujimoto et al., 2012; Lu et al., 2014; Nori et al., 2011; Uezono et al., 2018). However, the functional recovery obtained by cell transplantation alone is still limited, and the establishment of a more effective therapeutic method is desirable (Fujimoto et al., 2012; Lu et al., 2014; Nori et al., 2011). Several factors are thought to limit recovery: poor survival of the transplanted cells and poor axonal outgrowth of the transplanted cells due to the unfavorable environment in the injured spinal cord. After SCI, myelin debris and glial scars produce ligands of Nogo receptor-1 (NgR1), such as Nogo (Chen et al., 2000; GrandPre et al., 2000; Prinjha et al., 2000), myelin-associated glycoprotein (MAG) (McKerracher et al., 1994; Mukhopadhyay et al., 1994; Savio and Schwab, 1990), oligodendrocyte myelin glycoprotein (OMgp) (Wang et al., 2002), B lymphocyte stimulator (BLyS) (Zhang et al., 2009), and chondroitin sulfate proteoglycans (CSPGs) (Dickendesher et al., 2012). These ligands bind to NgR1 and cause growth cone collapse by activating the Ras homolog gene family member A (Rho-A) and Rho-associated kinase (ROCK), thereby inhibiting neuronal regeneration (Dergham et al., 2002; Fournier et al., 2003; Niederost et al., 2002). Blocking the Rho-ROCK cascade that is upregulated by NgR1 activation, therefore, was postulated to be beneficial in the treatment of SCI (Forgione and Fehlings, 2014; Wu and Xu, 2016).

Lateral olfactory tract usher substance (LOTUS), also known as cartilage acidic protein-1B (Crtac1B) (Sato et al., 2011), has been reported to function as an endogenous antagonist of NgR1. LOTUS binds to NgR1 and inhibits all of the ligand proteins (Nogo-A, MAG, OMgp, BLys, and CSPGs). As a result, growth cone collapse is reduced, and neurite outgrowth is promoted (Kawakami et al., 2018; Kurihara et al., 2014). As shown in a recent study, pan neuronal LOTUS overexpression under control of the mouse synapsin-1 promoter enhances the regeneration of raphespinal tract fibers in dorsal hemisection model transgenic mice, thereby contributing to the recovery of motor function (Hirokawa et al., 2017). We further verified the effects of LOTUS on a more clinically relevant contusive SCI model using the same mice. Overexpression of LOTUS in the spinal cord exerted neuroprotective and regenerative effects, which contributed to a significant restoration of motor function and nerve conduction after SCI (Ito et al., 2018). Given the beneficial effects of LOTUS on both neural tissue protection and regeneration, we hypothesized that the induction of LOTUS expression in NS/PCs before transplantation would produce an even more beneficial outcome by increasing the survival of transplanted cells and promoting axonal extension in SCI. Furthermore, LOTUS was also reported to suppress the axonal

This is an open access article under the CC BY license (http://creativecommons.org/licenses/by/4.0/).

growth-inhibiting receptor PIR-B, resulting in restraining growth cone collapse and neurite growth inhibition (Kurihara et al., 2020). PIR-B has been reported to function as a common receptor for Nogo, MAG, and OMgp, and inhibition of PIR-B activity partially restores neurite inhibition induced by these ligands (Atwal, 2008). Thus, LOTUS might exert stronger effects on axonal regeneration than NgR inhibitors by blocking both NgR1 and PIR-B.

In this study, we evaluated the efficacy of transplanting hiPSC-NS/PCs overexpressing LOTUS through lentiviral *ex vivo* gene transduction in subacute SCI. LOTUS suppressed the inhibition of axonal outgrowth and cellular apoptosis caused by NgR1 ligands and promoted the expression of neurotrophic factors in hiPSC-NS/PCs *in vitro*. Moreover, LOTUS enhanced the survival of graft cells and axonal extension in the injured spinal cord, contributing to further functional recovery. Thus, the combination of cell transplantation with *ex vivo* LOTUS gene transduction potentially represents a promising tool for the treatment of SCI.

RESULTS

Establishment of 414C2 hiPSC-NS/PCs expressing LOTUS via lentiviral infection

In this study, we first established LOTUS-expressing hiPSC-NS/PCs using lentiviral induction to evaluate whether the NgR1 antagonist LOTUS enhances the efficacy of neural stem cell transplantation in SCI. We generated a lentiviral vector encoding mouse LOTUS (mLOTUS) with a fluorescent protein marker (Venus) driven by an EF-1α promoter (CSII-EF1α-mLOTUS-IRES-Venus) (Figure 1A). The hiPSC-NS/PCs generated from 414C2-hiPSCs were infected with the LOTUS-expressing lentivirus and identified as LOTUS-NS/PCs. The control-NS/PCs were prepared by a similar transduction of ffLuc lentivirus without the mLOTUS coding sequence (Hara-Miyauchi et al., 2012). Fourth-passage neurospheres were used in both the *in vitro* and *in vivo* experiments.

Next, we performed real-time PCR analyses to determine the expression of the *Lotus* gene in the LOTUS-NS/PCs. Compared with the control cells, the LOTUS-NS/PCs expressed higher levels of the *Lotus* gene during both the neurosphere stage and its subsequent derivatives (Figure 1B). Western blotting analysis also showed the expression of the mLOTUS protein in the LOTUS-NS/PCs before and after differentiation (Figure 1C).

Changes in gene expression of the LOTUS-expressing NS/PCs

We performed real-time PCR to examine the effects of LOTUS on the differentiation profiles and secretion of trophic factors by LOTUS-overexpressing NS/PCs. The qPCR analysis of the differentiated cells on day 28 showed that LOTUS expression did not affect the expression of *NOGO* and *NGR1*, an inhibitor of axonal regeneration and its receptor, respectively (Figure S1A). Among the markers of cell differentiation, *MAP2* and *GFAP* were upregulated, while *OLIG2* and *NESTIN* expression was downregulated in the LOTUS-NS/PCs (Figure 1D). Comparable expression levels of *NESTIN*, an indicator of undifferentiated neural cells, were detected. Notably, the expression of neurotrophic factors, such as *BDNF*, *NT-3*, and *NGF*, was upregulated in the LOTUS-NS/PCs (Figure 1E). An NgR1 ligand stimulation assay, followed by real-time PCR was performed to investigate the effects of the presence of NgR1 ligands, such as Nogo, MAG, and OMgp, on the expression of neurotrophic factors. At 14 days after seeding on the wells precoated with PBS and each NgR1 ligand, both control-NS/PCs and LOTUS-NS/PCs showed no significant change in the expression of each neurotrophic factor in the presence of NgR1 ligands. Therefore, the upregulated expression of neurotrophic factors may be an effect of LOTUS expression but not NgR1 signaling (Figure S2A).

The LOTUS-expressing NS/PCs suppressed NgR1 ligand-mediated axonal inhibition and apoptosis *in vitro*

LOTUS acts as an NgR1 antagonist and inhibits downstream RhoA/ROCK signaling, suggesting that LOTUS expression in NS/PCs may promote axonal outgrowth and suppress cellular apoptosis (Ito et al., 2018). To verify this, we performed immunocytochemistry following a 2-week differentiation of the control- and LOTUS-NS/PCs. We measured the neurite length in the MAP2-positive cells and found that significantly longer dendrites were observed in the LOTUS-NS/PCs than in the control-NS/PCs (Figures 2A and 2B).

To determine whether LOTUS expression suppresses the inhibitory action of NgR1 ligands on neurite outgrowth, we performed a neurite outgrowth assay using the control-NS/PCs and the LOTUS-NS/PCs. The neurospheres of both NS/PC groups were dissociated into single cells and seeded on dishes precoated with PBS, Nogo, MAG, or OMgp. The lengths of the extended neurons were measured at 2 and 14 days after differentiation. In the presence of Nogo, MAG, or OMgp, the length of the neurite outgrowth was significantly shorter than that in PBS in the control-NS/PCs. However, there was no significant inhibition of axonal outgrowth on these ligand coatings in the LOTUS-NS/PCs at 2 and 14 days after seeding (Figures 2C and 2D). These results suggest that LOTUS prevented the inhibition of neurite outgrowth as an antagonist of NgR1.

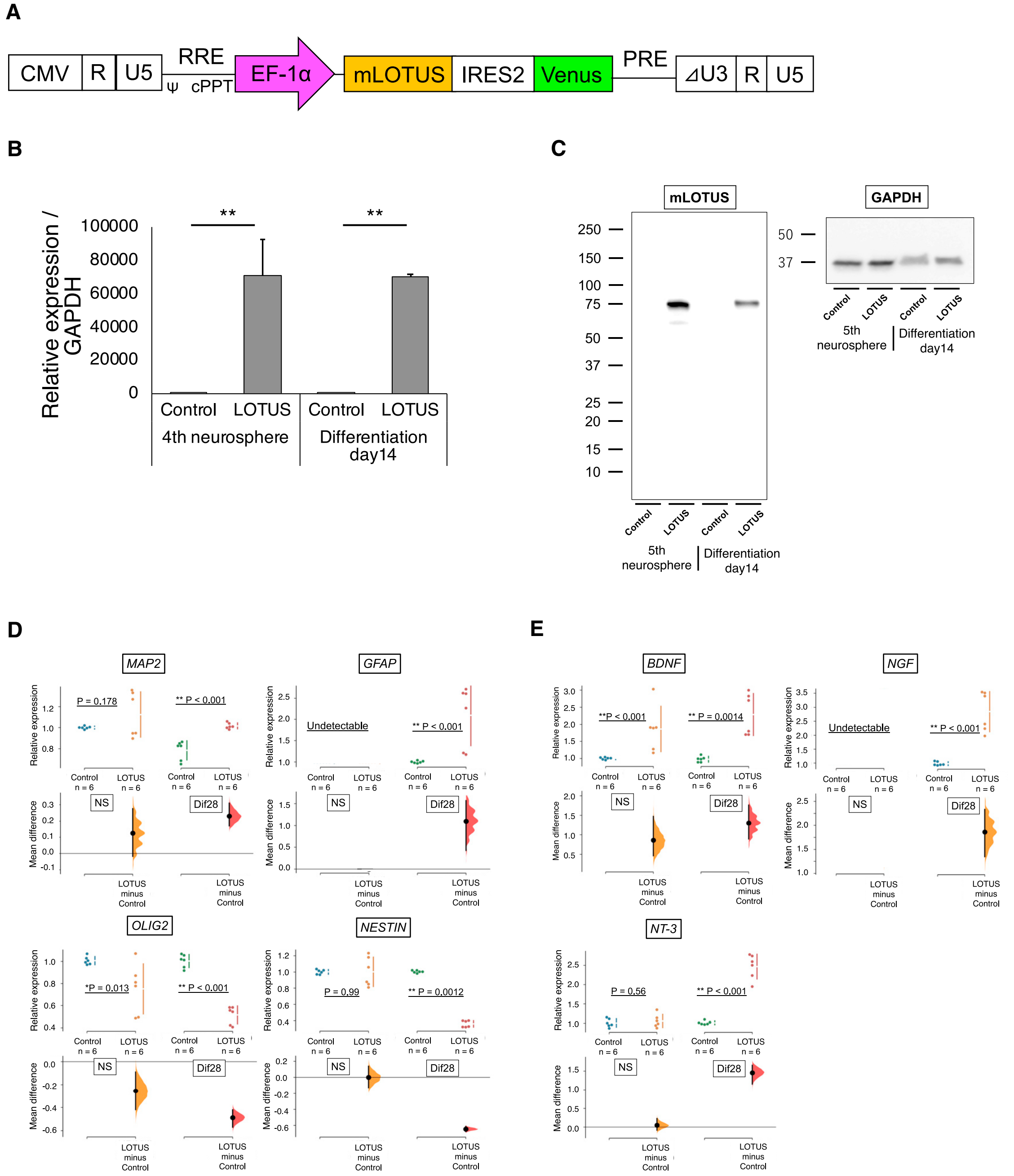

Figure 1. Mouse LOTUS (mLOTUS) expression in neurospheres and differentiated cells derived from 414C2 hiPSC-NS/PCs and gene expression change of LOTUS-expressing NS/PCs in the differentiation marker and neurotrophic factors

(A) Schematic illustration of the lentiviral vector CSII-EF1α-mLOTUS-IRES-Venus, which expresses mLOTUS cDNA and Venus fluorescent protein gene connected by an internal ribosomal entry site (IRES) under the control of EF1α promoter.

(B) Representative images of the neurosphere derived from control-NS/PCs and LOTUS-NS/PCs visualized by fluorescence microscopy.

(C) Western blot analyses of mLOTUS protein expression in both NS/PCs (LOTUS-NS/PCs; n = 5 independent experiments, control-NS/PCs; n = 5 independent experiments).

(D) Quantitative real-time PCR analyses for the gene expression of *MAP2*, *GFAP*, *OLIG2*, and *NESTIN* in both NS/PC groups (LOTUS-NS/PCs; n = 6 independent experiments, control-NS/PCs; n = 6 independent experiments).

(legend continued on next page)

We further examined the induction of apoptotic cell death by Nogo in the NS/PCs with or without LOTUS overexpression. The dissociated single cells of both NS/PCs were plated on dishes precoated with PBS or Nogo, and the apoptotic cells were counted 2 days post-plating. Immunostaining of cleaved caspase-3, a marker for apoptosis, revealed significantly fewer apoptotic cells in the LOTUS-NS/PCs than in the control-NS/PCs. Furthermore, the addition of Nogo significantly increased the apoptotic cells in the control-NS/PCs, whereas the induction of apoptosis was suppressed even in the presence of Nogo in the LOTUS-NS/PCs (Figures 2E and 2F).

The LOTUS-NS/PCs showed enhanced survival after transplantation and good differentiation into the three neural lineages

Previous *in vitro* data indicated that LOTUS suppresses cell apoptosis, promotes the secretion of trophic factors, and enhances axonal outgrowth in NS/PCs. We first examined the efficacy of LOTUS on promoting the survival of NS/PCs transplanted into the injured spinal cord by conducting bioluminescence imaging (BLI), as described in previous reports (Itakura et al., 2015; Kojima et al., 2019; Okada et al., 2005; Uezono et al., 2018). The BLI analysis revealed that the photon counts of the transplanted control-NS/PCs and LOTUS-NS/PCs and that the cells survived for up to 49 days after transplantation (Figures 3A and 3B). The engraftment rate of the transplanted LOTUS-NS/PCs reached 14.7% from the starting point to 49 days after transplantation, which was higher than 10.6% for the control-NS/PCs (Figure 3A). The histological analysis performed on day 54 revealed the presence of transplanted cells around the scar area that had migrated rostrally and caudally (Figure 3C).

We performed a histological analysis using H&E staining at 54 days after transplantation to evaluate the protective and regenerative effects of transplantation of LOTUS-expressing cells on the injured spinal cord (Figure 3D). Quantitative analyses revealed a significantly larger cross-sectional area of the spinal cord at the injury epicenter and 4 mm caudal to the epicenter in the LOTUS group than in the PBS group (Figure 3E), while no significant difference was observed between the areas in the control group and the PBS group.

Next, we evaluated the differentiation characteristics and tumorigenic risk of the transplanted cells using immunohistochemistry. Both the control-NS/PCs and the LOTUS-NS/PCs were differentiated into three neural lineages: ELAVL3/4-positive mature neurons, GFAP-positive astrocytes, and APC-positive oligodendrocytes (Figure 3F). Quantitative analyses revealed the differentiation rate of the transplanted cells. The proportions of ELAVL3/4-positive cells (control group: 48.6% ± 5.1%; LOTUS group: 58.6% ± 4.9%), GFAP-positive cells (control group: 22.4% ± 2.9%; LOTUS group: 24.6% ± 2.0%), and APC-positive cells (control group: 12.6% ± 2.2%; LOTUS group: 12.4% ± 0.6%) were not significantly different between the control group and the LOTUS group (Figure 3G). We also detected Ki-67+ and Nestin+ cells (Figure 4F). Immunostaining for various cell markers was examined 89 days after transplantation and subjected to quantitative analyses to evaluate the differentiation phenotype of the transplanted cells *in vivo*. Furthermore, Oct4-positive undifferentiated hiPSCs were not detectable and the percentages of Ki67+ proliferating cells (control group, 3.1% ± 0.7%; LOTUS group, 1.6% ± 0.3%) and Nestin+ cells (control group, 7.5% ± 2.2%; LOTUS group, 7.4% ± 0.6%) were low in each group without a significant difference, indicating that LOTUS did not increase the risk of tumorigenesis (Figure 3G).

The transplanted LOTUS-NS/PCs extensively spread into the injured spinal cord, differentiated into nerve fibers, and formed synaptic connections

We performed immunohistological analyses to identify the localization of the migrated LOTUS-NS/PCs at the caudal site of the injury, which is considered important for the assessment of the descending motor pathway. The immunostaining of STEM121 antibody, which is specific to human cytoplasm, in the axial section 4 mm caudal to the injury showed that the grafted LOTUS-NS/PCs spread to the dorsal and ventral columns, anterior and posterior horn, and central canal (Figures 4A–4G). The STEM121-positive fibers derived from the transplanted cells were co-stained with neurofilament 200 kDa (NF-H), suggesting that these fibers differentiated into neurofilament-positive neuronal axons (Figure 4H). Finally, triple immunostaining of HNA (a human-specific nuclear antigen), β-tubulin-3 and mouse-specific Bassoon (Bsn), a presynaptic marker, was performed to determine whether the differentiated transplanted LOTUS-NS/PCs were incorporated into the host neural circuit. The β-tubulin-3 and HNA double-positive cells that were differentiated from the LOTUS-NS/PCs were in contact with the Bsn-positive synaptic boutons of the host neurons (Figure 4I). Furthermore, triple immunostaining of HNA, β-tubulin-3, and human-specific synaptophysin (hSyn) demonstrated that HNA-negative and β-tubulin-3-positive host neural cells were in contact with

(E) Quantitative real-time PCR analyses for the gene expression of *BDNF*, *NGF*, and *NT-3* in both NS/PC groups (LOTUS-NS/PCs; n = 6 independent experiments, control-NS/PCs; n = 6 independent experiments). Values are the mean ± SEM; *p < 0.05, **p < 0.01. Values are the mean ± SEM; *p < 0.05, **p < 0.01. Statistical analysis was performed using the Mann-Whitney U test in each real-time PCR analysis.

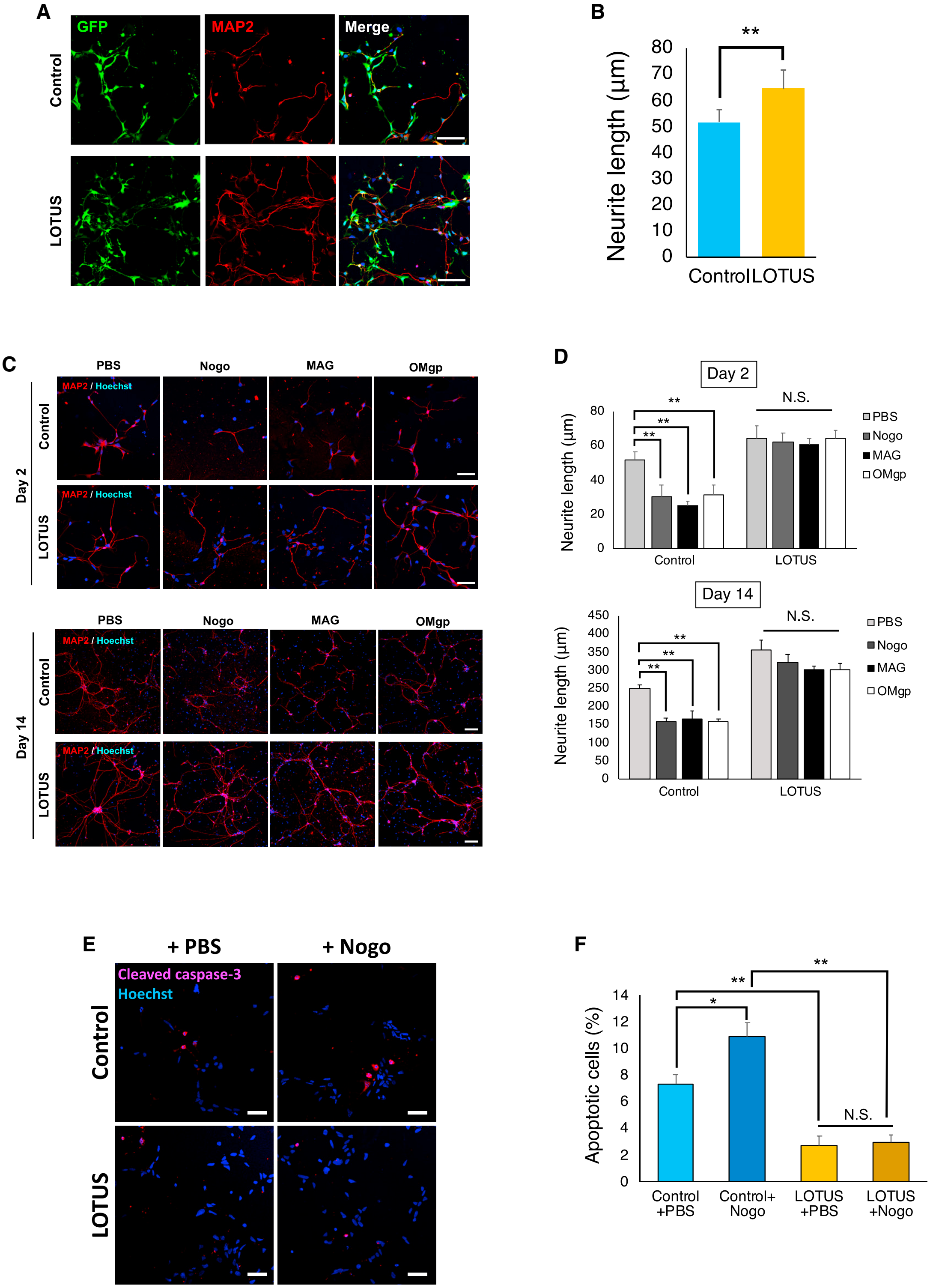

Figure 2. The LOTUS-expressing NS/PCs suppressed the axonal outgrowth inhibition extension and apoptosis *in vitro*
(A) Representative images of immunostaining for GFP and MAP2 at 2 days after differentiation.
(B) Quantitative analysis of the neurite length in both NS/PCs (LOTUS-NS/PCs; n = 5 independent experiments, control-NS/PCs; n = 5 independent experiments).

(legend continued on next page)

the hSyn-positive boutons (Figure 4J). These results suggest synaptic interactions between the graft-derived neurons and host neuronal cells.

LOTUS enhanced axonal elongation of graft cells into the injured spinal cord and promoted regrowth of raphespinal serotonergic fibers

To examine whether LOTUS promotes axonal outgrowth of transplanted cells in the injured spinal cord, we performed a detailed histological evaluation using STEM121 immunostaining. An enlarged image of STEM121 immunostaining showed axonal elongation of the transplanted cells of NS/PCs that had migrated from the lesion epicenter to the rostral and caudal site on the ventral aspect of the spinal cord (Figures 5A–5H). The LOTUS-NS/PCs showed more neuronal fibers derived from the transplanted cells than the control-NS/PCs in all images (rostral, epicenter, and caudal), and quantitative analysis revealed that a significantly larger STEM121-positive area was observed on the epicenter, rostral, and caudal sides (Figure 5I). These results suggested that LOTUS promoted neuronal elongation of grafted cells in the injured spinal cord.

To determine the regrowth of neurofilament and raphespinal serotonergic fibers (which are thought to contribute to the recovery of motor function after SCI in rodents) (Kaneko et al., 2006; Kim et al., 2004), we performed immunoreactions with the NF-H antibody in the mid-sagittal sections and the 5-hydroxytrytamine (5-HT) antibody in the axial sections at 4 mm caudal to the epicenter in the PBS, control, and LOTUS groups. Histological analyses demonstrated that cell transplantation caused an increase in the NF-H fibers or 5-HT-positive raphespinal serotonergic fibers in the raphe nucleus at the lumbar enlargement in all groups (Figures 5J and 5L). Quantitative analysis revealed that the LOTUS group had significantly greater NF-H or 5-HT-positive areas than the PBS and control groups (Figures 5K and 5M).

LOTUS enhanced the recovery of motor function following cell transplantation

Recovery of motor function following cell transplantation or PBS injection was assessed using the Basso mouse scale (BMS) scoring system, the rotarod test, and DigiGait footprint analysis. The BMS scores in the LOTUS group were significantly improved by 12 and 40 days after transplantation in the PBS group and control group, respectively (Figure 6A). Furthermore, the LOTUS group had a significantly higher BMS score than the control group at the final follow-up (Figure 6A). The LOTUS group also exhibited a significantly longer total run time in the rotarod test 54 days after transplantation than the PBS group (Figure 6B). Although the times were generally better in the LOTUS group than the control group, there was no significant difference (Figure 6B). In the treadmill gait analyses, using the DigiGait system, we observed a significantly longer hindlimb stride length and narrower hindlimb stance angle in the LOTUS group than the PBS group 54 days following transplantation. Regarding stride length, the LOTUS group showed a significantly greater improvement than the control group (Figure 6C). Phase dispersion, an indicator of coordination measured using DigiGait analysis, demonstrated that the LOTUS group exhibited significantly better improvements than the PBS group in diagonal and hindlimb coordination. A significantly improved diagonal coordination was also observed in the LOTUS group than the control group (Figure 6D). These transplanted cell-derived neurons were tentatively suggested to form synaptic connections with host neurons, leading to significant improvements in motor function. Since the *ex vivo* gene transduction of LOTUS in hiPSC-NS/PCs further enhances the effects following transplantation, this could be a promising adjunct in the treatment of subacute SCI with cell transplantation.

DISCUSSION

In this study, we evaluated the efficacy of transplanting hiPSC-NS/PCs transduced with the mLOTUS gene via lentivirus transduction in the treatment of subacute SCI. *In vitro*, the LOTUS-NS/PCs showed an increased neurite length compared with the control-NS/PCs after differentiation. LOTUS-NC/PCs also suppressed the inhibition of axonal outgrowth caused by NgR1 ligands and Nogo-induced cellular apoptosis. RT-PCR analyses revealed the upregulated gene expression of neurotrophic factors, such as *BDNF*, *NGF*, and *NT-3*, in the LOTUS-NS/PCs. *In vivo*, the LOTUS-NS/PCs showed increased engraftment and

(C) Representative images of neurite outgrowth inhibition due to Nogo, MAG, and OMgp, compared with PBS as control at 2 and 14 days post-differentiation.
(D) Quantitative analysis of the neurite length on the NgR ligands compared with PBS (LOTUS-NS/PCs; n = 5 independent experiments, control-NS/PCs; n = 5 independent experiments, for each ligand).
(E) Representative images of apoptotic cells immunostained with anti-cleaved caspase-3 antibody at 2 days post-differentiation.
(F) Quantitative analysis of the percentage of cleaved caspase-3-positive apoptotic cells (LOTUS-NS/PCs; n = 5 independent experiments, control-NS/PCs; n = 5 independent experiments). Values are the mean ± SEM; *$p < 0.05$, **$p < 0.01$. Statistical analysis was performed using Mann-Whitney U test in (B) and one-way ANOVA followed by the Tukey-Kramer test in (D and F). Scale bars, 50 μm in (A and E) and day 2 in (C), 100 μm day 14 in (C).

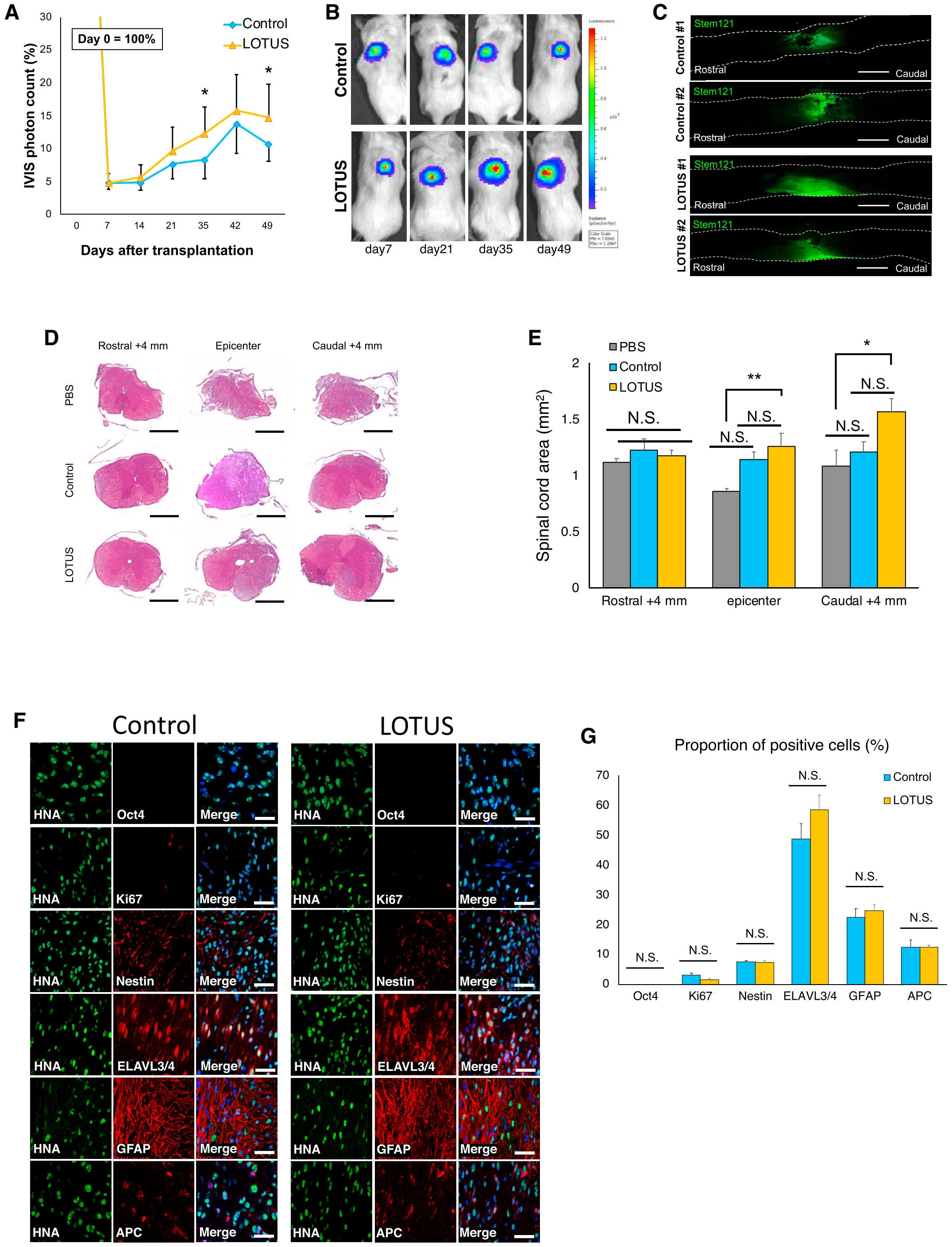

Figure 3. ***In vivo*** **imaging analyses of changes in the transplanted cells and histological analyses of spinal cord volume and differentiation profile**

(A) Quantitative analyses of the IVIS photon counts of the transplanted cells as percentage changes compared with the day of transplantation (LOTUS group; n = 8, control group; n = 6).

(B) Representative images of the IVIS photon counts up to 49 days.

(C) Representative images of immunostaining for STEM121 in the sagittal sections 54 days after transplantation.

(D) Representative images of H&E staining of the axial sections at 4 mm rostral, epicenter and 4 mm rostral to the epicenter.

(E) Quantitative analyses of the spinal cord area (LOTUS group; n = 5, control group; n = 5, PBS group n = 5).

(legend continued on next page)

survival following transplantation and protected the injured spinal cord tissues while successfully differentiating into all three lineages. Importantly, in the LOTUS group, the transplanted cells significantly extended their neuronal fibers to the rostral and caudal sites in the host spinal cord with significant regrowth of raphespinal serotonergic fibers, which are known to be responsible for functional recovery after the SCI in the rodents (Kaneko et al., 2006). These transplanted cell-derived neurons are tentatively suggested to form synaptic connections with host neurons, leading to significant improvements in motor function. Since the *ex vivo* gene transduction of LOTUS in hiPSC-NS/PCs further enhances the effects following transplantation, this could be a promising adjunct in the treatment of subacute SCI with cell transplantation.

In this study, LOTUS functioned as an NgR1 antagonist, resulting in the suppression of axonal outgrowth inhibition, even in the presence of NgR1 ligands *in vitro*. Consequently, these effects contributed to significant axonal outgrowth in the injured spinal cord after cell transplantation. NgR1 is suggested to play a critical role in axonal regeneration after SCI. Numerous studies have demonstrated and suggested that the blocking of NgR1 and its downstream signals should be considered when treating SCI. For instance, depressing NgR1 activation by administering NEP1-40, a Nogo-66 antagonist, led to axonal outgrowth with functional recovery after hemisection SCI (GrandPre et al., 2002; Li and Strittmatter, 2003). Similar results were observed using an SCI model of NgR1-deficient mice (Kim et al., 2004). Furthermore, the Rho inhibitor C3 transferase, which inhibits the downstream cascade of NgR1, resulted in axonal regrowth with recovery of locomotor function (Wu and Xu, 2016). Recently, Kurihara et al. (2020) reported that NgR1 antagonist LOTUS also suppressed axonal growth-inhibiting receptor PIR-B, resulting in restraining growth cone collapse and axonal growth inhibition. PIR-B has been reported as a common receptor for Nogo, MAG, and OMgp, and interfering with PIR-B activity partially rescued neurite inhibition by these ligands (Atwal, 2008). Thus, LOTUS could exert a stronger role for axonal regeneration than NgR1 inhibitors through blocking both NgR1 and PIR-B. In addition to the beneficial impact of LOTUS on host neural tissue, this study showed its effects on neurite extension in the transplanted cells themselves. Therefore, induction of the LOTUS gene for NS/PCs can be a beneficial option for improving neuronal regeneration.

NgR1 is known to initiate neuronal apoptosis by activating the Rho-A and ROCK pathways after SCI (Zhang et al., 2007). According to previous reports, NEP1-40 and Rho inhibitors suppress neuronal apoptosis in injury models of the central nervous system (CNS) (Dubreuil et al., 2003; Wang et al., 2008; Zhang et al., 2007). Our previous study also reported a neuroprotective effect of LOTUS on a transgenic mouse model of SCI, including a reduction in the number of apoptotic cells and preservation of the spinal tract and myelination (Ito et al., 2018). We therefore recognize that inhibition of apoptosis itself is one of the effects of LOTUS on SCI. In this study, we demonstrate the prevention of neuronal apoptosis in NS/PCs expressing LOTUS *in vitro*, and these results led to a significant increase in cell survival and host tissue protection after transplantation. Thus, inhibition of apoptosis by expressing LOTUS in NS/PCs was probably attributed to further functional restoration after SCI.

RT-PCR analysis showed that LOTUS upregulated the expression of the neurotrophic factors *BDNF*, *NGF*, and *NT-3* in the NS/PCs. These factors are already known for their positive attributes following transplantation therapy for CNS injuries. Lu et al. evaluated the efficacy of *BDNF* gene transfection in bone marrow stromal cells (MSCs), which were transplanted into the injured spinal cord. The transplanted BDNF-overexpressing MSCs promoted host axonal extension into the grafted cells (Lu et al., 2005). Another study demonstrated that BDNF-hypersecreting human MSCs enhanced the regrowth of host corticospinal and raphespinal tract fibers after SCI with significantly improved locomotor function (Sasaki et al., 2009). Kamei et al. showed the effectiveness of not only BDNF but also NGF and NT-3 secreted from NS/PCs and cocultured with organoids using the neonatal brain cortex and spinal cord. Transplantation of these cells achieved axonal sprouting of the corticospinal tract in organoids, but administration of antibodies against each trophic factor canceled this sprouting (Kamei et al., 2007). Together, these results from previous studies allowed us to hypothesize the favorable outcomes in this study: the three trophic factors potentially contribute to axonal outgrowth and functional recovery after SCI.

In this study, LOTUS antagonized NgR1 and upregulated neurotrophic factors, which facilitated the axonal elongation of grafted NS/PCs. These NS/PCs differentiated into nerve fibers such as neurofilament, contributing to the

(F) Representative images of immunostaining for each group at the injury site. HNA-positive transplanted cells were stained with Ki67, Nestin, HuC/HuD, GFAP, and APC. Nuclei were stained with Hoechst.
(G) The percentages of each marker-positive cells among the HNA-positive transplanted cells at 54 days after transplantation (LOTUS group; n = 5, control group; n = 5). Scale bars, 1,000 μm in (C), 500 μm in (D), and 20 μm in (F). Values are the mean ± SEM; $*p < 0.05$, $**p < 0.01$. Statistical analysis was performed using one-way ANOVA followed by the Tukey-Kramer test in (E) and Mann-Whitney U test in (G).

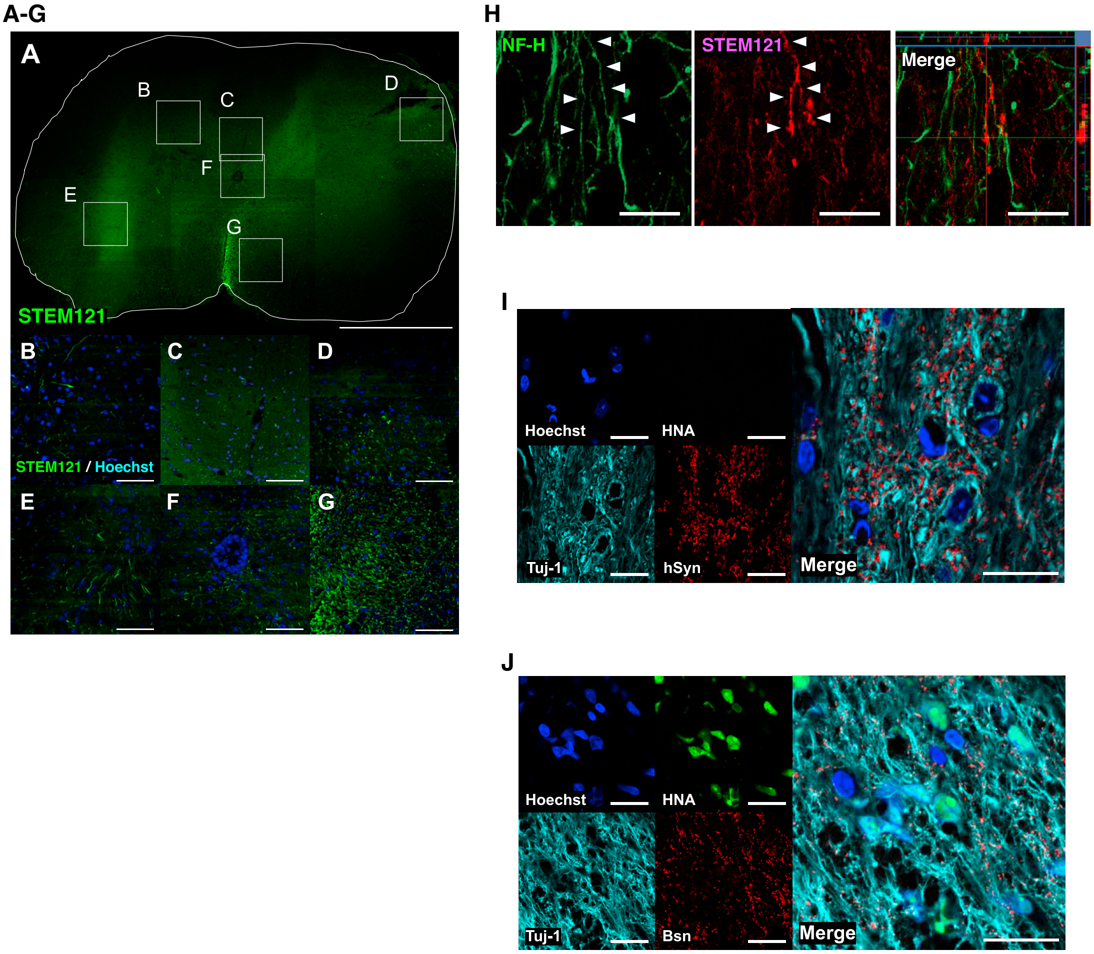

Figure 4. The transplanted LOTUS-NS/PCs were migrated and differentiated into the serotonergic neuronal fibers, and formed synaptic connection

(A) Representative images of immunostaining for STEM121 at the axial sections at 4 mm rostral to the epicenter in the LOTUS group.
(B–G) Enlarged images of the area in the white box in (A); posterior horn in (B), dorsal funiculus in (C), dorsal funiculus in (D), anterior horn in (E), central canal in (F), and ventral funiculus in (G).
(H) Representative images of immunostaining for STEM121 and NF-H at the sagittal sections in the LOTUS group.
(I) Representative images of immunostaining for HNA, β-tubulin-3, and Bassoon (Bsn) (mouse presynaptic marker). HNA/β-tubulin-3 double-positive neurons, which were derived from transplanted cells, contacted Bsn-positive host cells.
(J) Representative images of immunostaining for HNA, β-tubulin-3 and hSyn (human-specific presynaptic marker). hSyn-positive boutons contacted HNA-negative and β-tubulin-3-positive host mouse neurons.
Scale bars, 500 μm in (A), 50 μm in (B–G, I, and J), and 20 μm in (H).

increase in raphespinal fibers in the lumbar enlargement of the spinal cord. Although the raphespinal tract is recognized for its modulatory effects on sensory activity (Mason, 2001), it can function as an alternative circuit, connecting to the motor circuit after SCI (Liang et al., 2015). In fact, a previous study using the serotonergic neurotoxin 5,7-dihydro-xytryptamine demonstrated that raphespinal regeneration following SCI significantly contributed to functional restoration in NgR1-deficient mice (Kim et al., 2004). Similarly, our previous data showed raphespinal regeneration with functional recovery using LOTUS-overexpressing transgenic mice (Ito et al., 2018). These results supported the theory that increased raphespinal serotonergic fibers were relevant to locomotor recovery.

We transduced LOTUS into hiPSC-NS/PCs *ex vivo* using lentivirus and successfully improved the results gained through transplantation therapy. Zhilai et al. (2011) reported that transplantation of MSCs expressing NEP1-40 resulted in significant functional recovery following SCI, but this treatment only affected axonal regeneration in the host cells and

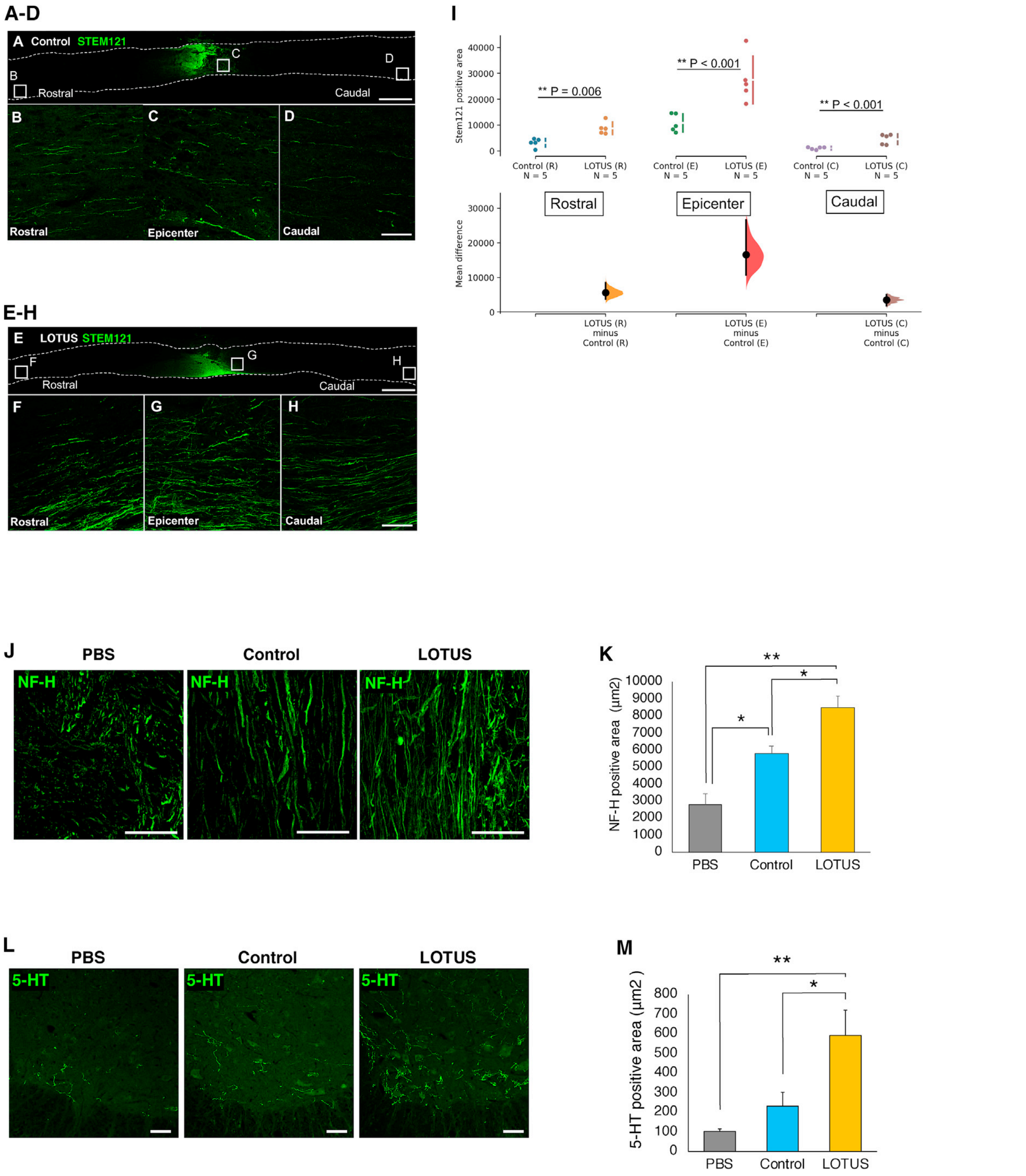

Figure 5. LOTUS enhanced axonal extension of the transplanted cells and serotonergic neuronal regeneration in the injured spinal cord

(A) Representative images of immunostaining for STEM121 at the sagittal sections in the control group.

(B–D) Enlarged images of the area in the white box in (A); rostral in (B), epicenter in (C), and caudal in (D).

(E) Representative images of immunostaining for STEM121 at the sagittal sections in the LOTUS group.

(F–H) Enlarged images of the area in the white box in (E); rostral in (F), epicenter in (G), and caudal in (H).

(I), Quantitative analysis of the STEM121-positive area (LOTUS group; n = 5, control group; n = 5).

(J) Representative images of immunostaining for NF-H at mid-sagittal sections at 4 mm caudal to the epicenter.

(K) Quantitative analysis of NF-H-positive area (LOTUS group; n = 5, control group; n = 5, PBS group; n = 5).

(legend continued on next page)

not in the transplanted cells. On the other hand, our LOTUS-expressing NS/PCs contributed to the improved survival and increased axonal outgrowth of the transplanted cells by LOTUS expressing into themselves, and further affect host neurons by acting as a source of LOTUS secretion. How far the secreted LOTUS diffuses in the injured spinal cord remains to be verified. When soluble LOTUS was injected into the vitreous of the eye, it reached the frustrated tip of the optic nerve by diffusion (Kawakami et al., 2018), thus it is possible that LOTUS diffused into the host neuron in this study as well. In the injured spinal cord, the expression of LOTUS was halved (Hirokawa et al., 2017; Ito et al., 2018), and conversely, the expression of NgR1 and its ligands of such as Nogo were increased (Hunt et al., 2003). The delivery of LOTUS via *ex vivo* gene transfer with cell transplantation led to synergistic effects by replacing the lost neurons in the injured area with transplanted iPSCs and exogenously replenishing the decreased LOTUS. Furthermore, the heightened expression of neurotrophic factors strongly enhanced this synergistic effect, resulting in the advantage in the treatment of SCI. In other clinical fields, *ex vivo* gene therapy is expected to be applied to gene deficiency and CNS diseases (Akhtar et al., 2018). Clinical research for the treatment of β-thalassemia has already been approved using gene therapy with autologous CD34+ cells transduced with BB305 (lentivirus) vector (Thompson et al., 2018). Therefore, *ex vivo* gene therapy could be a clinically feasible strategy for the treatment of SCI.

In this study, we established hiPSC-NS/PCs expressing LOTUS and a system to transplant them into an SCI mouse model. On the other hand, in this experiment, we were unable to easily provide evidence that inhibition of NgR1 signaling by LOTUS expression in hiPSC-NS/PCs directly induced improved functional recovery after SCI. However, *in vitro*, Takei and coworkers showed that LOTUS binds to NgR1 and blocks NgR1 ligand binding to its receptor (Kurihara et al., 2012, 2014). Therefore, LOTUS suppresses NgR1-induced inhibition of axonal elongation and growth cone collapse (Kurihara et al., 2014). Similar to these results, the current *in vitro* analysis also showed that axon elongation was not inhibited in LOTUS-overexpressing NS/PCs, even in the presence of NgR1 ligands (Figure 2C). Therefore, based on the *in vitro* results, we estimated that LOTUS contributed to axonal elongation and restored locomotor function by inhibiting NgR1 signaling. In fact, *in vivo* effects of the blockade of NgR1 signaling in inducing the functional recovery after SCI are obvious based on the previous studies, which reported that a hemisection SCI model of NgR1 knockout mice (Kim et al., 2004) or SCI mice treated with an NgR1 antagonist showed axonal extension and motor functional recovery following injury (Li and Strittmatter, 2003). One of the possible solutions to clarify the roles of transplanted LOTUS-NS/PCs *in vivo* in future studies would be to use NgR1 knockout mice as a model of SCI and transplant NS/PCs. If no NgR1-independent action of LOTUS is observed because NgR1 signaling is eliminated in this mouse model, then the therapeutic effect of NS/PCs expressing LOTUS exogenously on locomotor function would be the same as that in the control-NS/PC group (without exogenous LOTUS expression). On the other hand, if the therapeutic effects of exogenous LOTUS-expressing NS/PCs are stronger than those of control-NS/PCs, then some NgR1-independent action of exogenous LOTUS expression should be detected.

In conclusion, we showed that the transduction of LOTUS resulted in a significantly increased axonal extension, reduced apoptosis, and increased neurotrophic factor secretion *in vitro* and enhanced the survival of transplanted cells and the regrowth of raphespinal serotonergic fibers with synaptic formation integrated into the host neural circuitry. We believe that these results contribute to the recovery of motor function following SCI. *Ex vivo* gene therapy delivering the NgR1 antagonist LOTUS with cell transplantation can represent a therapeutic strategy for SCI.

EXPERIMENTAL PROCEDURES

Lentiviral vector preparation

Lentiviral vector production and infection of cells were performed as described previously (Miyoshi et al., 1998; Okada et al., 2005). Mouse LOTUS (mLOTUS) cDNA and the Venus fluorescent protein (Nagai et al., 2002; Duran-Avelar et al., 2018) gene connected by an internal ribosomal entry site (IRES) were cloned into the lentiviral vector CSII-EF1α, yielding pCSII-EF1α-mLOTUS-IRES-Venus (Figure 1A) (Iida et al., 2017). Recombinant lentiviral vector production and titer determination were performed as described previously (Hashizume et al., 2015).

Cell culture, lentivirus transduction, and neuronal differentiation analysis

The cell culture, neural induction, and neuronal differentiation analysis of hiPSCs (414C2) were performed as described previously (Itakura et al., 2015) with slight modifications. In brief, the hiPSC-NS/PCs were dissociated and infected with the mLOTUS-expressing lentivirus and a lentivirus expressing ffLuc, a fusion protein between Venus fluorescent protein and firefly luciferase (Hara-Miyauchi et al., 2012) under the control of the EF1α promoter, which were prepared

(L) Representative images of immunostaining for 5-HT at the axial sections at 4 mm caudal to the epicenter.
(M) Quantitative analysis of 5-Ht-positive area (LOTUS group; n = 5, control group; n = 5, PBS group; n = 5).
Scale bars, 1,000 μm in (A and E), 100 μm in (B–D and F–H), and 50 μm in (J and L). Values are the mean ± SEM; *p < 0.05, **p < 0.01. Statistical analysis was performed using Mann-Whitney U test in (I) and one-way ANOVA followed by the Tukey-Kramer test in (K and M).

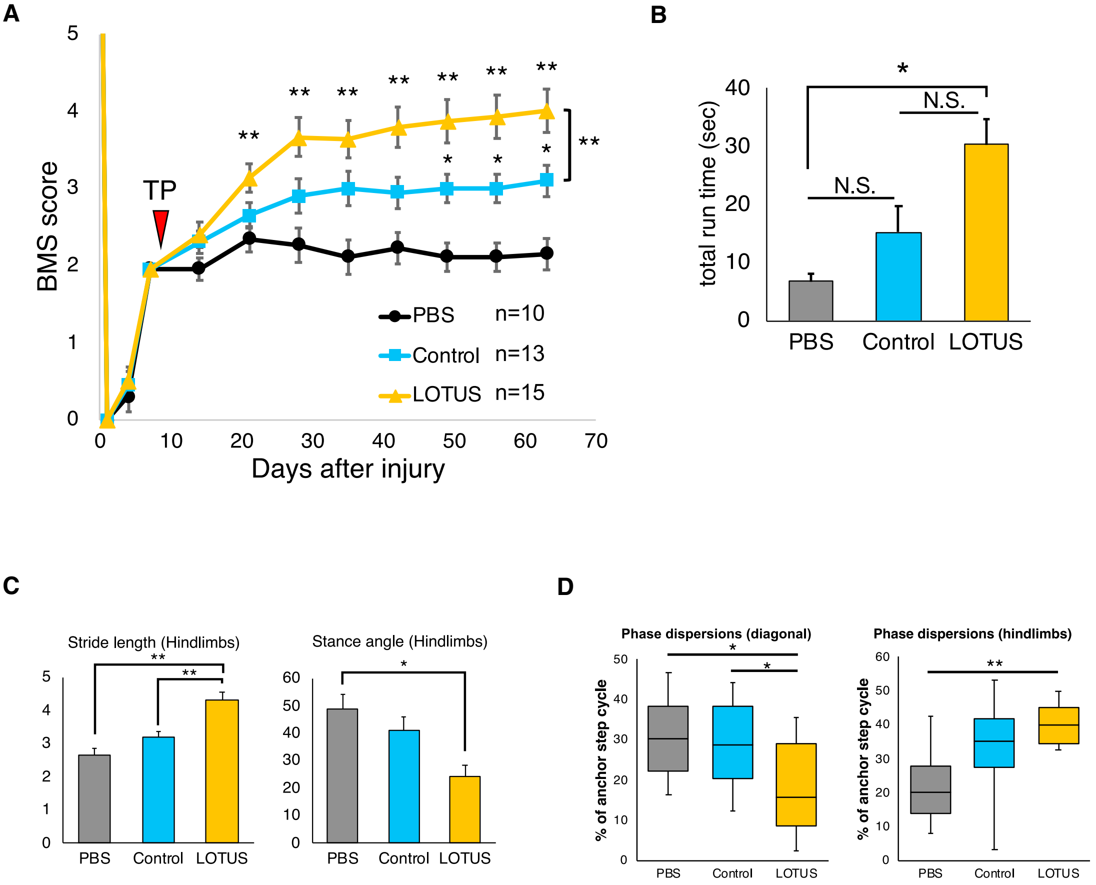

Figure 6. Evaluation of motor functional recovery

(A) Hindlimb motor function was evaluated weekly for 63 days after SCI (54 days after transplantation) by BMS scores in the LOTUS group, the control group and the PBS group (LOTUS group; n = 15, control group; n = 13, PBS group; n = 10).

(B) The rotarod test was performed 54 days after transplantation, and quantitatively analysis of total run time was evaluated (LOTUS group; n = 15, control group; n = 13, PBS group; n = 10).

(C) Treadmill gait analyses using a DigiGait system were examined 54 days after transplantation, and quantitative analyses of the stride length and stance angle were performed (LOTUS group; n = 15, control group; n = 13, PBS group; n = 10).

(D) Hindlimb coordination was analyzed by phase dispersions as the diagonal/hindlimbs coordination in DigiGait analyses (LOTUS group; n = 15, control group; n = 13, PBS group; n = 10). Values are the mean ± SEM; $*p < 0.05$, $**p < 0.01$. Statistical analysis was performed using two-way repeated-measures ANOVA with Tukey's test in the analysis of the BMS score and one-way ANOVA followed by the Tukey-Kramer test in the rotarod test and DigiGait analyses.

as LOTUS-NS/PCs. In contrast, the control-NS/PCs were prepared by similar transduction via lentivirus expressing ffLuc. Detailed methods are presented in supplemental experimental procedures.

Western blotting

Protein isolation and western blotting were performed to determine the protein expression of the control-NS/PCs and the LOTUS-NS/PCs. Detailed methods are presented in supplemental experimental procedures.

Real-time PCR

RNA extraction and real-time PCR were performed as described previously (Nori et al., 2011), and detailed protocols are provided in supplemental experimental procedures.

Neurite outgrowth assay on NgR1 ligand-coated plates

Neurite outgrowth assay on NgR1 ligand-coated plates was performed for the evaluation of MAP2-positive neurite length *in vitro*. Detailed methods are presented in supplemental experimental procedures.

Apoptosis analysis

Immunostaining with anti-cleaved caspase-3 antibody was performed for the evaluation of cellular apoptosis *in vitro*. Detailed methods are presented in supplemental experimental procedures.

Animals

We used adult female NOD-SCID (NOD/ShiJic-scidJcl mice, 18–22 g; Oriental Yeast, Tokyo, Japan) in this study. A total of 47 mice (18

mice in the LOTUS group, 17 in the control group, and 12 in the PBS group) were used in the experiment, 9 of which died during follow-up (3 mice in the LOTUS group, 4 in the control group, and 2 in the PBS group). All experiments were approved by the ethics committee of Keio University and were in accordance with the Guide for the Care and Use of Laboratory Animals (National Institutes of Health, Bethesda, MD).

SCI and transplantation

Contusive SCI was induced at the 10th thoracic level in the spinal cords of adult female NOD-SCID mice. Nine days after the injury, LOTUS-NS/PCs or control-NS/PCs (5×10^5 cells/2 μL) were transplanted into the lesion epicenter of each mouse. Detailed methods are presented in supplemental experimental procedures.

In vivo imaging of transplanted cells

BLI was performed as described previously (Itakura et al., 2015). Detailed methods are presented in supplemental experimental procedures.

Immunohistochemistry

Immunohistochemistry (IHC) analysis was performed for the *in vitro* evaluation. Detailed methods are presented in supplemental experimental procedures.

Quantitative analysis of the tissue sections

Quantitative analysis of the tissue sections following SCI and transplantation was performed. Detailed methods are presented in supplemental experimental procedures.

Behavioral analysis

The locomotor function of each mouse in the control group, the LOTUS group, and the PBS group was evaluated weekly using the BMS up to 63 days after injury (Basso et al., 2006). Detailed methods are presented in supplemental experimental procedures.

Statistical analysis

All data are presented as the mean ± SEM. The Mann-Whitney U test for single comparisons was used to detect any significant differences between groups with respect to the RT-PCR analyses and the IHC results. One-way ANOVA followed by the Tukey-Kramer test for multiple comparisons was used to evaluate the differences in the neurite outgrowth assay, apoptosis analyses, H&E staining, IHC results, rotarod test, and DigiGait results. Repeated-measures two-way ANOVA followed by the Tukey-Kramer test was used for the BMS analyses and IVIS photon counts. Differences were considered significant at $p < 0.05$ or $p < 0.01$. IBM SPSS Statistics (v.24) was used for all calculations. In the Gardner-Altman plots, we used DABEST (data analysis with bootstrap-coupled estimation): open-source libraries for MATLAB, Python, and R (https://www.estimationstats.com) (Ho et al., 2019).

SUPPLEMENTAL INFORMATION

Supplemental information can be found online at https://doi.org/10.1016/j.stemcr.2021.09.006.

AUTHOR CONTRIBUTIONS

S.I. designed the project, performed most of the experiments, collected and interpreted the data, and wrote the manuscript, with technical assistance from N.N. Y.K., K.K., S.N., M.M., and K.T. provided experimental support and ideas for the project. M.N. and H.O. designed the studies, supervised the overall project, and prepared the final manuscript.

ACKNOWLEDGMENTS

We thank S. Yamanaka at CiRA (Kyoto University) for the human iPSCs (414C2). We are grateful for the assistance of H. Miyoshi, T. Okubo, Y. Hoshino, Y. Tanimoto, R. Shibata, K. Kajikawa, K. Ago, T. Kitagawa, and M. Kawai, who are all members of the spinal cord research team at the Department of Orthopedic Surgery and Physiology, Keio University School of Medicine, Tokyo, Japan.We also thank T. Harada, K. Yasutake, and M. Akizawa for their assistance with the experiments and animal care. This work was supported by the Japan Agency for Medical Research and Development (AMED) grant JP JJ P21bm0204001 (to H.O. and M.N.) and partly by The General Insurance Association of Japan Medical Research Grant on traffic accidents 16-1-20.

Received: March 13, 2021
Revised: September 13, 2021
Accepted: September 14, 2021
Published: October 14, 2021

REFERENCES

Akhtar, A.A., Gowing, G., Kobritz, N., Savinoff, S.E., Garcia, L., Saxon, D., Cho, N., Kim, G., Tom, C.M., Park, H., et al. (2018). Inducible expression of GDNF in transplanted iPSC-derived neural progenitor cells. Stem Cell Reports *10*, 1696–1704.

Atwal, J.K. (2008). PirB is a functional receptor for myelin inhibitors of axonal regeneration. Science. 10.1126/science.1161151.

Basso, D.M., Fisher, L.C., Anderson, A.J., Jakeman, L.B., McTigue, D.M., and Popovich, P.G. (2006). Basso mouse scale for locomotion detects differences in recovery after spinal cord injury in five common mouse strains. J. Neurotrauma *23*, 635–659.

Chen, M.S., Huber, A.B., van der Haar, M.E., Frank, M., Schnell, L., Spillmann, A.A., Christ, F., and Schwab, M.E. (2000). Nogo-A is a myelin-associated neurite outgrowth inhibitor and an antigen for monoclonal antibody IN-1. Nature *403*, 434–439.

Dergham, P., Ellezam, B., Essagian, C., Avedissian, H., Lubell, W.D., and McKerracher, L. (2002). Rho signaling pathway targeted to promote spinal cord repair. J. Neurosci. *22*, 6570–6577.

Dickendesher, T.L., Baldwin, K.T., Mironova, Y.A., Koriyama, Y., Raiker, S.J., Askew, K.L., Wood, A., Geoffroy, C.G., Zheng, B., Liepmann, C.D., et al. (2012). NgR1 and NgR3 are receptors for chondroitin sulfate proteoglycans. Nat. Neurosci. *15*, 703–712.

Dubreuil, C.I., Winton, M.J., and McKerracher, L. (2003). Rho activation patterns after spinal cord injury and the role of activated Rho in apoptosis in the central nervous system. J. Cell Biol. *162*, 233–243.

Duran-Avelar, M.J., Vazquez-Reyes, A., Gonzalez-Mercado, A.L., Zambrano-Zaragoza, J.F., Ayon-Perez, M.F., Agraz-Cibrian, J.M., Gutierrez-Franco, J., and Vibanco-Perez, N. (2018). pirA- and pirB-like gene identification in *Micrococcus luteus* strains in Mexico. J. Fish Dis. *41*, 1667–1673.

Forgione, N., and Fehlings, M.G. (2014). Rho-ROCK inhibition in the treatment of spinal cord injury. World Neurosurg. *82*, e535–e539.

Fournier, A.E., Takizawa, B.T., and Strittmatter, S.M. (2003). Rho kinase inhibition enhances axonal regeneration in the injured CNS. J. Neurosci. *23*, 1416–1423.

Fujimoto, Y., Abematsu, M., Falk, A., Tsujimura, K., Sanosaka, T., Juliandi, B., Semi, K., Namihira, M., Komiya, S., Smith, A., and Nakashima, K. (2012). Treatment of a mouse model of spinal cord injury by transplantation of human induced pluripotent stem cell-derived long-term self-renewing neuroepithelial-like stem cells. Stem Cells *30*, 1163–1173.

GrandPre, T., Li, S., and Strittmatter, S.M. (2002). Nogo-66 receptor antagonist peptide promotes axonal regeneration. Nature *417*, 547–551.

GrandPre, T., Nakamura, F., Vartanian, T., and Strittmatter, S.M. (2000). Identification of the Nogo inhibitor of axon regeneration as a Reticulon protein. Nature *403*, 439–444.

Hara-Miyauchi, C., Tsuji, O., Hanyu, A., Okada, S., Yasuda, A., Fukano, T., Akazawa, C., Nakamura, M., Imamura, T., Matsuzaki, Y., et al. (2012). Bioluminescent system for dynamic imaging of cell and animal behavior. Biochem. Biophys. Res. Commun. *419*, 188–193.

Hashizume, O., Ohnishi, S., Mito, T., Shimizu, A., Ishikawa, K., Nakada, K., Soda, M., Mano, H., Togayachi, S., Miyoshi, H., et al. (2015). Corrigendum: epigenetic regulation of the nuclear-coded GCAT and SHMT2 genes confers human age-associated mitochondrial respiration defects. Sci. Rep. *5*, 14591.

Hirokawa, T., Zou, Y., Kurihara, Y., Jiang, Z., Sakakibara, Y., Ito, H., Funakoshi, K., Kawahara, N., Goshima, Y., Strittmatter, S.M., and Takei, K. (2017). Regulation of axonal regeneration by the level of function of the endogenous Nogo receptor antagonist LOTUS. Sci. Rep. *7*, 12119.

Ho, J., Tumkaya, T., Aryal, S., Choi, H., and Claridge-Chang, A. (2019). Moving beyond P values: data analysis with estimation graphics. Nat. Methods *16*, 565–566.

Hunt, D., Coffin, R.S., Prinjha, R.K., Campbell, G., and Anderson, P.N. (2003). Nogo-A expression in the intact and injured nervous system. Mol. Cell Neurosci. *24*, 1083–1102.

Iida, T., Iwanami, A., Sanosaka, T., Kohyama, J., Miyoshi, H., Nagoshi, N., Kashiwagi, R., Toyama, Y., Matsumoto, M., Nakamura, M., and Okano, H. (2017). Whole-genome DNA methylation analyses revealed epigenetic instability in tumorigenic human iPS cell-derived neural stem/progenitor cells. Stem Cells *35*, 1316–1327.

Itakura, G., Kobayashi, Y., Nishimura, S., Iwai, H., Takano, M., Iwanami, A., Toyama, Y., Okano, H., and Nakamura, M. (2015). Controlling immune rejection is a fail-safe system against potential tumorigenicity after human iPSC-derived neural stem cell transplantation. PLoS One *10*, e0116413.

Ito, S., Nagoshi, N., Tsuji, O., Shibata, S., Shinozaki, M., Kawabata, S., Kojima, K., Yasutake, K., Hirokawa, T., Matsumoto, M., et al. (2018). LOTUS inhibits neuronal apoptosis and promotes tract regeneration in contusive spinal cord injury model mice. eNeuro *5*. https://doi.org/10.1523/ENEURO.0303-18.2018.

Kamei, N., Tanaka, N., Oishi, Y., Hamasaki, T., Nakanishi, K., Sakai, N., and Ochi, M. (2007). BDNF, NT-3, and NGF released from transplanted neural progenitor cells promote corticospinal axon growth in organotypic cocultures. Spine *32*, 1272–1278.

Kaneko, S., Iwanami, A., Nakamura, M., Kishino, A., Kikuchi, K., Shibata, S., Okano, H.J., Ikegami, T., Moriya, A., Konishi, O., et al. (2006). A selective Sema3A inhibitor enhances regenerative responses and functional recovery of the injured spinal cord. Nat. Med. *12*, 1380–1389.

Kawakami, Y., Kurihara, Y., Saito, Y., Fujita, Y., Yamashita, T., and Takei, K. (2018). The soluble form of LOTUS inhibits nogo receptor-mediated signaling by interfering with the interaction between nogo receptor type 1 and p75 neurotrophin receptor. J. Neurosci. *38*, 2589–2604.

Kim, J.E., Liu, B.P., Park, J.H., and Strittmatter, S.M. (2004). Nogo-66 receptor prevents raphespinal and rubrospinal axon regeneration and limits functional recovery from spinal cord injury. Neuron *44*, 439–451.

Kojima, K., Miyoshi, H., Nagoshi, N., Kohyama, J., Itakura, G., Kawabata, S., Ozaki, M., Iida, T., Sugai, K., Ito, S., et al. (2019). Selective ablation of tumorigenic cells following human induced pluripotent stem cell-derived neural stem/progenitor cell transplantation in spinal cord injury. Stem Cells Transl. Med. *8*, 260–270.

Kurihara, Y., Takai, T., and Takei, K. (2020). Nogo receptor antagonist LOTUS exerts suppression on axonal growth-inhibiting receptor PIR-B. J. Neurochem. *155*, 285–299.

Kurihara, Y., Iketani, M., Ito, H., Nishiyama, K., Sakakibara, Y., Goshima, Y., and Takei, K. (2014). LOTUS suppresses axon growth inhibition by blocking interaction between Nogo receptor-1 and all four types of its ligand. Mol. Cell Neurosci. *61*, 211–218.

Kurihara, Y., Arie, Y., Iketani, M., Ito, H., Nishiyama, K., Sato, Y., Nakamura, F., Mizuki, N., Goshima, Y., and Takei, K. (2012). The carboxyl-terminal region of Crtac1B/LOTUS acts as a functional domain in endogenous antagonism to Nogo receptor-1. Biochem. Biophys. Res. Commun. *418*, 390–395.

Li, S., and Strittmatter, S.M. (2003). Delayed systemic Nogo-66 receptor antagonist promotes recovery from spinal cord injury. J. Neurosci. *23*, 4219–4227.

Liang, H., Wang, S., Francis, R., Whan, R., Watson, C., and Paxinos, G. (2015). Distribution of raphespinal fibers in the mouse spinal cord. Mol. Pain *11*, 42.

Lu, P., Jones, L., and Tuszynski, M. (2005). BDNF-expressing marrow stromal cells support extensive axonal growth at sites of spinal cord injury. Exp. Neurol. *191*, 344–360.

Lu, P., Woodruff, G., Wang, Y., Graham, L., Hunt, M., Wu, D., Boehle, E., Ahmad, R., Poplawski, G., Brock, J., et al. (2014). Long-distance axonal growth from human induced pluripotent stem cells after spinal cord injury. Neuron *83*, 789–796.

Mason, P. (2001). Contributions of the medullary raphe and ventromedial reticular region to pain modulation and other homeostatic functions. Annu. Rev. Neurosci. *24*, 737–777.

McKerracher, L., David, S., Jackson, D.L., Kottis, V., Dunn, R.J., and Braun, P.E. (1994). Identification of myelin-associated glycoprotein as a major myelin-derived inhibitor of neurite growth. Neuron *13*, 805–811.

Miyoshi, H., Blomer, U., Takahashi, M., Gage, F.H., and Verma, I.M. (1998). Development of a self-inactivating lentivirus vector. J. Virol. *72*, 8150–8157.

Mukhopadhyay, G., Doherty, P., Walsh, F.S., Crocker, P.R., and Filbin, M.T. (1994). A novel role for myelin-associated glycoprotein as an inhibitor of axonal regeneration. Neuron *13*, 757–767.

Nagai, T., Ibata, K., Park, E.S., Kubota, M., Mikoshiba, K., and Miyawaki, A. (2002). A variant of yellow fluorescent protein with fast and efficient maturation for cell-biological applications. Nat. Biotechnol. *20*, 87–90.

Niederost, B., Oertle, T., Fritsche, J., McKinney, R.A., and Bandtlow, C.E. (2002). Nogo-A and myelin-associated glycoprotein mediate neurite growth inhibition by antagonistic regulation of RhoA and Rac1. J. Neurosci. *22*, 10368–10376.

Nori, S., Okada, Y., Yasuda, A., Tsuji, O., Takahashi, Y., Kobayashi, Y., Fujiyoshi, K., Koike, M., Uchiyama, Y., Ikeda, E., et al. (2011). Grafted human-induced pluripotent stem-cell-derived neurospheres promote motor functional recovery after spinal cord injury in mice. Proc. Natl. Acad. Sci. U S A *108*, 16825–16830.

Okada, S., Ishii, K., Yamane, J., Iwanami, A., Ikegami, T., Katoh, H., Iwamoto, Y., Nakamura, M., Miyoshi, H., Okano, H.J., et al. (2005). In vivo imaging of engrafted neural stem cells: its application in evaluating the optimal timing of transplantation for spinal cord injury. FASEB J. *19*, 1839–1841.

Prinjha, R., Moore, S.E., Vinson, M., Blake, S., Morrow, R., Christie, G., Michalovich, D., Simmons, D.L., and Walsh, F.S. (2000). Inhibitor of neurite outgrowth in humans. Nature *403*, 383–384.

Sasaki, M., Radtke, C., Tan, A.M., Zhao, P., Hamada, H., Houkin, K., Honmou, O., and Kocsis, J.D. (2009). BDNF-hypersecreting human mesenchymal stem cells promote functional recovery, axonal sprouting, and protection of corticospinal neurons after spinal cord injury. J. Neurosci. *29*, 14932–14941.

Sato, Y., Iketani, M., Kurihara, Y., Yamaguchi, M., Yamashita, N., Nakamura, F., Arie, Y., Kawasaki, T., Hirata, T., Abe, T., et al. (2011). Cartilage acidic protein-1B (LOTUS), an endogenous Nogo receptor antagonist for axon tract formation. Science *333*, 769–773.

Savio, T., and Schwab, M.E. (1990). Lesioned corticospinal tract axons regenerate in myelin-free rat spinal cord. Proc. Natl. Acad. Sci. U S A *87*, 4130–4133.

Thompson, A.A., Walters, M.C., Kwiatkowski, J., Rasko, J.E.J., Ribeil, J.-A., Hongeng, S., Magrin, E., Schiller, G.J., Payen, E., Semeraro, M., et al. (2018). Gene therapy in patients with transfusion-dependent beta-thalassemia. N. Engl. J. Med. *378*, 1479–1493.

Uezono, N., Zhu, Y., Fujimoto, Y., Yasui, T., Matsuda, T., Nakajo, M., Abematsu, M., Setoguchi, T., Mori, S., Takahashi, H.K., et al. (2018). Prior treatment with anti-high mobility group box-1 antibody boosts human neural stem cell transplantation-mediated functional recovery after spinal cord injury. Stem Cells *36*, 737–750.

Wang, K.C., Koprivica, V., Kim, J.A., Sivasankaran, R., Guo, Y., Neve, R.L., and He, Z. (2002). Oligodendrocyte-myelin glycoprotein is a Nogo receptor ligand that inhibits neurite outgrowth. Nature *417*, 941–944.

Wang, Q., Gou, X., Xiong, L., Jin, W., Chen, S., Hou, L., and Xu, L. (2008). Trans-activator of transcription-mediated delivery of NEP1-40 protein into brain has a neuroprotective effect against focal cerebral ischemic injury via inhibition of neuronal apoptosis. Anesthesiology *108*, 1071–1080.

Wu, X., and Xu, X.M. (2016). RhoA/Rho kinase in spinal cord injury. Neural Regen. Res. *11*, 23–27.

Zhang, L., Zheng, S., Wu, H., Wu, Y., Liu, S., Fan, M., and Zhang, J. (2009). Identification of BLyS (B lymphocyte stimulator), a non-myelin-associated protein, as a functional ligand for Nogo-66 receptor. J. Neurosci. *29*, 6348–6352.

Zhang, Y., Gu, X., and Yuan, X. (2007). Phenylalanine activates the mitochondria-mediated apoptosis through the RhoA/Rho-associated kinase pathway in cortical neurons. Eur. J. Neurosci. *25*, 1341–1348.

Zhilai, Z., Hui, Z., Yinhai, C., Zhong, C., Shaoxiong, M., Bo, Y., and Anmin, J. (2011). Combination of NEP 1-40 infusion and bone marrow-derived neurospheres transplantation inhibit glial scar formation and promote functional recovery after rat spinal cord injury. Neurol. India *59*, 579–585.

Stem Cell Reports, Volume *16*

Supplemental Information

LOTUS overexpression via *ex vivo* gene transduction further promotes recovery of motor function following human iPSC-NS/PC transplantation for contusive spinal cord injury

Shuhei Ito, Narihito Nagoshi, Yasuhiro Kamata, Kota Kojima, Satoshi Nori, Morio Matsumoto, Kohtaro Takei, Masaya Nakamura, and Hideyuki Okano

Supplementary Information

LOTUS overexpression via lentiviral *ex vivo* gene transduction further promotes recovery of motor function following human iPSC-NS/PC transplantation for contusive spinal cord injury

Shuhei Ito, Narihito Nagoshi, Yasuhiro Kamata, Kota Kojima, Satoshi Nori, Morio Matsumoto, Kohtaro Takei, Masaya Nakamura, and Hideyuki Okano

Figure S1

A.

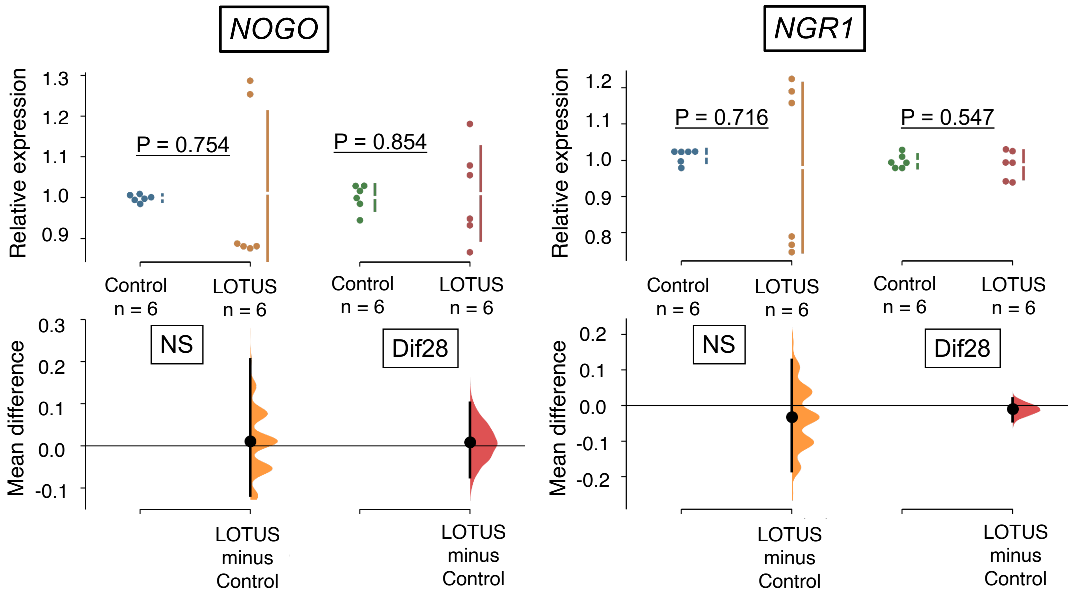

Figure S1

Gene expression change of LOTUS-expressing NS/PCs in *NOGO* and *NGR1* in neurospheres and differentiated cells derived from 414C2 hiPSC-NS/PCs.

***A*,** Quantitative real time PCR analyses for the gene expression of *NOGO* and *NGR1* in both NS/PC groups (LOTUS-NS/PCs; n = 6 independent experiments, Control-NS/PCs; n = 6 independent experiments). Values are the mean ± SEM; $*p < 0.05$, $**p < 0.01$. Statistical analysis was performed using the Mann–Whitney U test in each real time PCR analysis.

Figure S2

A.

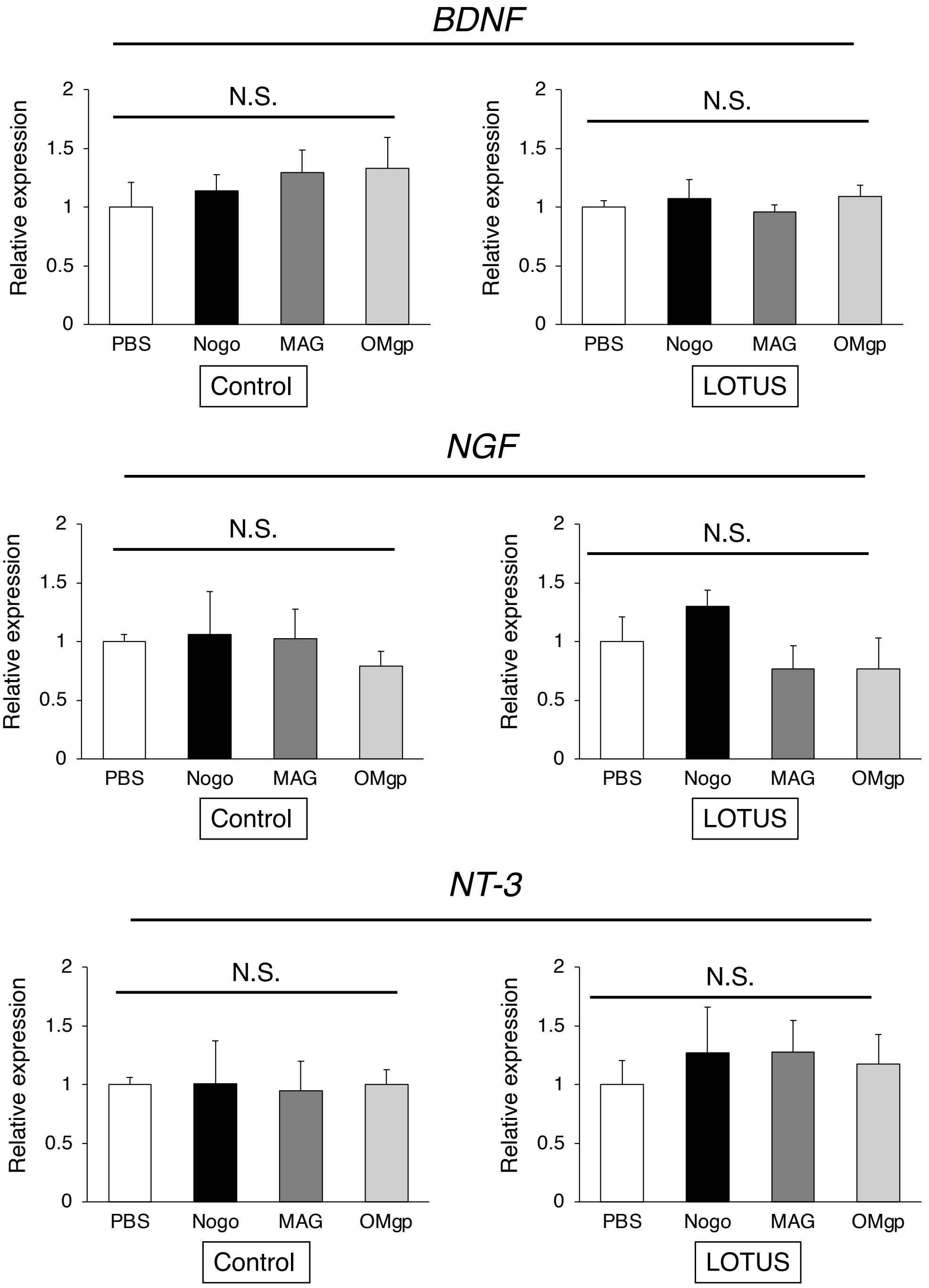

Figure S2

Gene expression change of the Control-NS/PCs and LOTUS-NS/PCs in the neurotrophic factors under the presence of NgR1 ligands.

A, Quantitative real time PCR analyses for the gene expression of *BDNF, NGF* and *NT-3* under precoated with PBS, Nogo, MAG or OMgp in both NS/PC groups (LOTUS-

NS/PCs; n = 4 independent experiments, Control-NS/PCs; n = 4 independent experiments). Values are the mean ± SEM; *$p < 0.05$, **$p < 0.01$. Statistical analysis was performed using one-way ANOVA followed by the Tukey-Kramer test in each real time PCR analysis.

Supplemental Experimental Procedures

Cell culture, lentivirus transduction, and neuronal differentiation analysis

The cell culture and neural induction of hiPSCs (414C2) were performed as previously described (Itakura et al., 2015) with slight modifications. The hiPSCs were cultured for 12 days on gelatin-coated (0.1%) culture dishes with mouse embryonic fibroblasts and then formed into embryo bodies in floating culture for 30 days. Aggregated cells were dissociated into single cells using TrypLE Select (Thermo Fisher Scientific, Yokohama, Japan) and cultured in media containing a hormone mixture supplemented with B27 and 20 ng/ml FGF-2 (Pepro Tech, Rocky Hill, NJ) and 10 ng/ml human leukemia inhibitory factor (hLIF; Merck KGaA, Darmstadt, Germany) for 12 days. These primary neurospheres were passaged to fourth neurospheres for the *in vitro* experiment and transplantation.

LOTUS-NS/PCs were prepared via lentivirus transduction. The 414C2-hiPSC-derived primary neurospheres were dissociated and infected with the mLOTUS-expressing lentivirus. Subsequently, these secondary neurospheres were dissociated and infected with lentivirus to express ffLuc, a fusion protein between Venus fluorescent protein and firefly luciferase (Hara-Miyauchi et al., 2012) under the control of the EF1α promoter (pCSII-EF1α-ffLuc). These secondary neurospheres were passaged into tertiary and fourth neurospheres and used for *in vitro* analysis and transplantation. In contrast, the Control-NS/PCs were prepared by similar transduction via lentivirus expressing ffLuc and used for the analyses.

Neuronal differentiation analysis was performed as follows. Dissociated hiPSC-NS/PCs were plated onto poly-D-lysin/laminin-coated 48-well slides (Costar 3548; Corning, NY, USA) at a density of 1×10^5 cells/ml and cultured in medium without growth factors at 37 °C in 5% CO_2 and 95% air for 2, 14 and 28 days. Differentiated cells were fixed with 4% paraformaldehyde (PFA) in 0.1 M phosphate buffered saline (PBS) for 15 minutes at RT and stained with the following primary antibodies for immunocytochemistry: anti-GFP (goat IgG, 1:500, Rockland, Ireland), anti-MAP2 (rabbit IgG, 1:500, Merck Millipore, Billerica, Massachusetts, USA) and anti-cleaved caspase-3 (rabbit IgG, 1:500, Cell Signaling, Beverly, MA, USA). Then, the sections were incubated with Alexa Fluor-conjugated secondary antibodies (1:1000). Nuclei were stained with Hoechst 33258 (10

µg/ml, Sigma-Aldrich, St. Louis, MO, USA). All *in vitro* images were obtained using confocal laser scanning microscopy (LSM 700; Carl Zeiss, Jena, Germany).

SCI and transplantation

Previous study (Nori et al., 2011) showed the sufficient functional recovery of BMS 4-5 following cell transplantation alone. In this study, we compared three groups: PBS, Control-NS/PCs and LOTUS-NS/PCs. Even though the efficacy of cell transplantation alone has been demonstrated, these experiments were conducted to show the efficacy of cell transplantation with ex-vivo gene transduction. To prevent ceiling effect of the locomotor function, we induced severer model of SCI with 65kdyn.

Adult female NOD-SCID mice were anesthetized by intraperitoneal injections of ketamine (60 mg/kg) and xylazine (10 mg/kg). The laminal arch of the vertebrae at the tenth thoracic level was removed, and the dorsal surface of the dura mater was exposed and subjected to a 65-kdyn contusive SCI using a commercially available SCI device (IH impactor, Precision Systems and Instrumentation) as previously described(Scheff et al., 2003). After SCI, 12.5 mg/kg ampicillin was administered intramuscularly. Nine days after the injury, 5×10^5 LOTUS-NS/PCs/2 µl were transplanted into the lesion epicenter of each mouse (LOTUS group; n = 18) using a metal needle at a rate of 1 µl/minute with a 10 µl Hamilton syringe and a stereotaxic microinjector (KDS 310; Muromachi Kikai, Tokyo, Japan). Using the same method, 5×10^5 Control-NS/PC/2 µl were injected into each mouse (control group; n = 17). An equal volume of PBS was injected into the PBS group of mice (n = 12).

Western blotting

We performed western blotting to determine the protein expression of the Control-NS/PCs and the LOTUS-NS/PCs. The fourth passage neurospheres that underwent differentiation for 14 days were used for analysis. Cell samples were homogenized in lysis buffer, and 10 ng protein was electrophoretically separated on 4–20% gradient polyacrylamide gels and transferred onto polyvinylidene difluoride (PVDF) membranes. The membranes were blocked with 5% skim milk in Tris-buffered saline and 0.1% Tween 20 (TBST) for 1 h at room temperature (RT) and then incubated overnight at 4 °C with an affinity-purified monoclonal mouse antibody against LOTUS (ITM). After the membranes were washed with TBST, they were incubated with horseradish peroxidase-

conjugated secondary antibody for 1 h at RT. The bands were visualized using an enhanced chemiluminescence reagent (GE Healthcare) and an ImageQuant LAS 4000 instrument (GE Healthcare).

Real-time PCR

Total RNA was extracted from hiPSC-NS/PCs (LOTUS-NS/PCs; n = 5, Control-NS/PCs; n = 5) by using an RNeasy Micro Kit (Qiagen, Inc., Hilgen, Germany), and cDNA was synthesized by reverse transcription with ReverTra Ace qPCR RT master mix (ToyoboCo., Ltd., Life Science Department, Osaka, Japan). Quantitative polymerase chain reaction (QT-PCR) was performed using Step One Plus™ (Applied Biosystems, Foster City, CA, USA) following the manufacturer's instructions. The expression levels of each gene were normalized to that of GAPDH using the ΔΔCT method. We chose primers used for the TaqMan gene expression assays and TaqMan gene expression 96-well fast plates (Life Technologies, Carlsbad, CA, USA). We used the following manufactured primers (Applied Biosystems) against mouse or human DNA sequences, such as *Lotus, MAP2, GFAP, OLIG2, NNESTIN, BDNF, NGF, NT-3 and GAPDH.*

Neurite outgrowth assay on NgR1 ligand-coated plates

NgR1 ligand-coated plates were prepared for the neurite outgrowth assay. Twenty-four-well chamber slides (Costar 3524; Corning, NY, USA) were coated with poly-D-lysin/laminin overnight at 37 °C in 5% CO2 and 95% air. These slides were washed with PBS and dried for 30 minutes. Then, Nogo-66-Fc (400 nM, R&D Systems), MAG-AP (300 nM, R&D Systems) or OMgp (200 nM, R&D Systems) was added to the slide and incubated at 37 °C for 2 h. After washing, these plates were coated with laminin overnight at 37 °C in 5% CO2 and 95% air. Dissociated hiPSC-NS/PCs were plated onto NgR1 ligand-coated plates at a density of $1.2{\times}10^4$ cells/ml and cultured in medium without growth factors at 37 °C in 5% CO2 and 95% air for 2 and 14 days. Immunostaining was performed with an anti-MAP2 antibody in blocking solution overnight at 4 °C, followed by incubation with Alexa555-labeled goat antibodies against rabbit IgG (1:1000 dilution, Invitrogen) in blocking solution for 1 h at RT. Nuclei were stained with Hoechst 33258. The images were obtained using a confocal laser scanning microscope (LSM 700; Carl Zeiss, Jena, Germany) with 20x objective lenses. We randomly obtained five regions in the culture slides, and then neurite length of the differentiated NS/PCs was measured by

manually tracing the length of the longest neurite per cell (using NIH Image software). Five longest neurites of each cell on each region were measured (n = 5 in the both hiPSC-NS/PCs).

Apoptosis analysis

Twenty-four-well chamber slides (Costar 3524; Corning, NY, USA) were coated with poly-D-lysin/laminin overnight at 37 °C in 5% CO2 and 95% air, followed by washing with PBS and drying for 30 minutes. Nogo-66-Fc (400 nM, R&D Systems) was added to the slide and incubated at 37 °C for 2 h. After washing, these plates were coated with laminin overnight at 37 °C in 5% CO2 and 95% air. Dissociated hiPSC-NS/PCs were plated onto the Nogo-coated plates at a density of 1.2×10^4 cells/ml and cultured in medium without growth factors at 37 °C in 5% CO2 and 95% air for 2 days. Immunostaining was performed with anti-cleaved caspase-3 in blocking solution overnight at 4°C, followed by incubation with Alexa555-labeled goat antibodies against rabbit IgG (1:1000 dilution, Invitrogen) in blocking solution for 1 h at RT. Nuclei were stained with Hoechst 33258. The images were obtained using confocal laser scanning microscopy (LSM 700) with 20x objective lenses. We randomly obtained five images in the culture slides,and then the cleaved caspase-3-positive apoptotic cells and nuclei were counted in each image.These counts were expressed as a percentage of the total nuclei (*n* =5, each group).

***In vivo* imaging of transplanted cells**

For confirmation of the survival of the transplanted hiPSC-NS/PCs, a Xenogen-IVIS spectrum cooled charge-coupled device (CCD) optical macroscopic imaging system (Caliper Life-Science, Hopkinton, MA, USA) was used for bioluminescence imaging (BLI). Monitoring was performed once per week following cell transplantation. Six mice in Control group and eight mice in LOTUS group were examined for *in vivo* imaging. In brief, D-luciferin (VivoGlo Luciferin; Promega, Madison, WI) was intraperitoneally injected at a dose of 300 mg/kg body weight. Animals were placed in a light-tight chamber, and photons emitted from luciferase-expressing cells were collected with integration times of 5 s to 2 minutes, depending on the intensity of bioluminescence emission. BLI signals were quantified in maximum radiance units (photons per second per centimeter squared per steradian (p/s/cm2/sr)) and are presented as log10 (photons per second) values.

Immunohistochemistry (IHC)

Anesthetized mice were transcardially perfused with heparinized saline solution, followed by 4% paraformaldehyde PBS 63 days after injury. Spinal cords were dissected and postfixed in 4% paraformaldehyde for 2 h at RT. Fixed spinal cords were soaked in 10% sucrose in 0.1 M PBS overnight at 4 °C, followed by 30% sucrose, embedding in Optimal Cutting Temperature compound (Sakura FineTechnical Co., Ltd.), and freezing as previously described(Nishimura et al., 2013). Samples were sectioned in the sagittal plane at a thickness of 14 µm or the axial plane at a thickness of 20 µm on a cryostat (Leica CM3050 S, Leica Microsystems). Histological analyses of the sections were performed by hematoxylin-eosin (HE) staining and IHC. Tissue sections were stained with the following primary antibodies for IHC: anti-STEM121 (mouse IgG1, 1:100; TaKaRa Bio, Y40410), anti-Oct4 (mouse IgG1, 1:50 Santa Cruz, sc-5279), anti-Ki67 (rabbit IgG, 1:200; Abcam, ab15580), anti- human Nestin (rabbit IgG, 1:200; Immuno-Biological Laboratories, Takasaki, Japan, 18741), anti-HuC/HuD (ELAVL3/4) (mouse IgG2, 1:100 Invitrogen, A21271), anti-glial fibrillary acidic protein (GFAP; rabbit IgG, 1:500; Dako, Z0334), anti-APC (mouse IgG2b, 1:300; Abcam, ab16794), anti-neurofilament 200 kDa (NF-H; mouse IgG, 1:500; Millipore, MAB5262), anti-5-HT (goat IgG, 1:500; Immunostar, Inc., 20079), anti-b-tubulin-3 (mouse IgG2b, 1:500), anti-nuclei (HNA, mouse IgG1; Sigma-Aldrich, 1:200, MAB4383), anti-Bsn (mouse IgG, 1:200; Stressgen, ADI-VAM-PS003), and anti-hSyn (mouse IgG, 1:200; Merck KGaA, MAB332). Then, the sections were incubated with Alexa Fluor-conjugated secondary antibodies (1:1000). Nuclei were stained with Hoechst 33258. All images were obtained using a confocal laser scanning microscope (LSM 700, Carl Zeiss).

Quantitative analysis of the tissue sections

The spinal cord area was quantified using HE staining of axial sections at the lesion epicenter and 4 mm rostral and caudal to the epicenter under a 10x objective (n = 5, each group). Quantitative analysis of the STEM121 positive fibers was performed using the immunoreactive areas in midsagittal sections at the lesion epicenter and 4 mm rostral and caudal to the epicenter under a 40x objective (n = 5, each group). To quantify the NF-H-positive fibers, the midsagittal sections were captured at 4 mm caudal to the epicenter under a 40x objective (n = 5, each group). To quantify the 5-HT-positive fibers,

we scanned the ventral horn of axial sections at 4 mm caudal to the epicenter (lumbar enlargement) under a 20x objective (*n* = 5, each group). To quantify the proportion of each cell phenotype in vivo, we selected representative midsagittal sections and randomly obtained five regions in the graft cells under a 20x objective (*n* = 5, each group). The numbers of marker-positive cells, such as HNA-, Oct4-, Ki67-, Nestin-, ELAVL3/4 (HuC/D)-, GFAP-, and APC-positive cells were counted in each section (n = 5 per group). All images were quantified using ImageJ (https://imagej.nih.gov/ij/). The threshold values were maintained at a constant level for all analyses using ImageJ.

Behavioral analysis

The hindlimb locomotor function of each mouse was evaluated weekly using the Basso mouse scale (BMS) up to 63 days after injury (54 days after transplantation)(Basso et al., 2006). The BMS scores was determined for 13 mice in Control group, 15 mice in LOTUS group and 10 mice in PBS group. Two persons blinded to the mouse group performed the behavioral analyses. At 63 days after injury, motor function was also assessed on a rotating rod(Rotarod)apparatus (KDS310; Muromachi-Kikai Co., Ltd.) by measuring the amount of time that mice could remain on the rod while it rotated at 10 rotations per minute (rpm). Three trials were conducted and the average number of seconds was recorded. Six mice each from the three groups were examined in the Rotarod test. A treadmill gait analysis was performed using the DigiGait System (Mouse Specifics). Ten mice each from control group and LOTUS group and 5 mice from PBS group were used. Three cycles of continuous gait were recorded and each parameters of each limb were measured. The stride lengths and stance angles of the hindlimbs were measured on a treadmill at a speed of 7 cm/s, and phase dispersion was analyzed in the DigiGait analyses to provide an indication of limb coordination.

Supplemental Reference

Nishimura S, Yasuda A, Iwai H, Takano M, Kobayashi Y, Nori S, Tsuji O, Fujiyoshi K, Ebise H, Toyama Y, Okano H, Nakamura M (2013) Time-dependent changes in the microenvironment of injured spinal cord affects the therapeutic potential of neural stem cell transplantation for spinal cord injury. Mol Brain 6:3.
